# Small Molecule Inhibitor Targeting CDT1/Geminin Protein Complex Promotes DNA Damage and Cell Death in Cancer Cells

**DOI:** 10.3389/fphar.2022.860682

**Published:** 2022-04-25

**Authors:** Nikolaos Karantzelis, Michalis Petropoulos, Valeria De Marco, David A. Egan, Alexander Fish, Evangelos Christodoulou, David W. Will, Joe D. Lewis, Anastassis Perrakis, Zoi Lygerou, Stavros Taraviras

**Affiliations:** ^1^ Department of Physiology, Medical School, University of Patras, Patras, Greece; ^2^ Department of General Biology, Medical School, University of Patras, Patras, Greece; ^3^ Division of Biochemistry, Netherlands Cancer Institute, Amsterdam, Netherlands; ^4^ Chemical Biology Core Facility, European Molecular Biology Laboratory, Heidelberg, Germany

**Keywords:** AlphaScreen, small molecule inhibitor, high-throughput screening, Geminin, CDT1, cancer

## Abstract

DNA replication initiation requires the loading of MCM2-7 complexes at the origins of replication during G1. Replication licensing renders chromatin competent for DNA replication and its tight regulation is essential to prevent aberrant DNA replication and genomic instability. CDT1 is a critical factor of licensing and its activity is controlled by redundant mechanisms, including Geminin, a protein inhibitor of CDT1. Aberrant CDT1 and Geminin expression have been shown to promote tumorigenesis *in vivo* and are also evident in multiple human tumors. In this study, we developed an *in vitro* AlphaScreen™ high-throughput screening (HTS) assay for the identification of small-molecule inhibitors targeting the CDT1/Geminin protein complex. Biochemical characterization of the most potent compound, AF615, provided evidence of specific, dose-dependent inhibition of Geminin binding to CDT1 both *in-vitro* and in cells. Moreover, compound AF615 induces DNA damage, inhibits DNA synthesis and reduces viability selectively in cancer cell lines, and this effect is CDT1-dependent. Taken together, our data suggest that AF615 may serve as a useful compound to elucidate the role of CDT1/Geminin protein complex in replication licensing and origin firing as well as a scaffold for further medicinal chemistry optimisation.

## Introduction

The accurate and timely duplication of the genome is vital for the preservation of genomic integrity. A crucial event during this process is the formation of a multiprotein complex at the origins of DNA replication through a process which is called licensing. From late mitosis until the end of G1 phase, thousands of origins are licensed through the loading of double hexamers of minichromosome maintenance 2–7 complexes (MCM2–7) (Symeonidou et al., 2012; Champeris Tsaniras et al., 2014; Parker et al., 2017). Τhe six-subunit origin recognition complex (ORC; subunits ORC1–6), the cell division cycle 10-dependent transcript 1 (CDT1), and the cell division cycle 6 (CDC6) are required for the loading of MCMs and together these factors form the chromatin-bound multiprotein complexes called pre-replicative complexes (pre-RCs). During G1/S transition pre-PRCs are converted to pre-initiation complexes (pre–ICs), with the subsequent origin firing and replication start (Siddiqui et al., 2013; Petropoulos et al., 2019).

To prevent origin licensing in S or G2 phase, strict regulation of CDT1 protein levels is crucial. Upon entry into S phase, CDT1 is targeted for ubiquitin-dependent proteolysis by multiple negative regulators (Nishitani et al., 2006; Zhou et al., 2020). In metazoans, Geminin binds to CDT1 during S and G2 phases, preventing untimely MCM loading onto chromatin (Patmanidi et al., 2017). Deregulated replication licensing has been linked to genomic instability, a well characterised hallmark of cancer (Halazonetis et al., 2008; Negrini et al., 2010; Macheret and Halazonetis, 2015). Reduced, ectopic, or increased origin licensing have been reported to induce replication stress, leading to under-replicated or re-replicated DNA which fuels genomic instability (Petropoulos et al., 2019). Aberrant expression of CDT1 causes DNA re-replication, activation of the damage response and apoptosis in human cancer cells (Vaziri et al., 2003). Furthermore, Geminin depletion leads to re-replication of the genome, DNA damage, mitotic abnormalities and genomic instability in cancer cells (Melixetian et al., 2004). Deregulation of CDT1 or Geminin protein levels has also been described to play a role in carcinogenesis. Prolonged CDT1 overexpression in pre-malignant cells, bypasses the antitumor barriers of senescence and apoptosis and promotes clonal expansion and malignant behaviour (Liontos et al., 2007). Moreover, transgenic mice overexpressing Cdt1 in thymocytes in a p53 mutant background are prone to lymphoblastic lymphomas (Seo et al., 2005) and mice overexpressing both Cdt1 and Cdc6 form dysplasias in the intestinal epithelium (Muñoz et al., 2017). In addition, mice lacking Geminin show enhanced tumorigenesis in the colon and lung epithelium (Champeris Tsaniras et al., 2018). Both CDT1 and Geminin are overexpressed in cancer cell lines as well as in several tumor types, even from the early stages of cancer progression (Xouri et al., 2004; Bravou et al., 2005; Liontos et al., 2007).

Deregulation of CDT1 or Geminin does not have the same impact in non-transformed cell lines or normal tissues. CDT1 overexpression does not induce any detectable DNA re-replication in non-transformed cells, in contrast to cancer cells which are driven to apoptosis (Tatsumi et al., 2006). Furthermore, Geminin depletion does not induce re-replication and cell death in non-transformed cells, as opposed to different cancer cell lines (Zhu and DePamphilis, 2009). Given the fact that CDT1 and Geminin activity relies on their direct protein-protein interaction, we suggest that chemical compounds targeting the CDT1/Geminin protein complex might serve as selective anticancer agents.

Here, we developed and applied an optimized AlphaScreen™ HTS assay in order to identify small-molecule inhibitors targeting the Geminin/CDT1 protein complex. In total, 23,000 compounds were screened and classified according to their inhibitory effect on Geminin/CDT1 interaction. Using a threshold based on statistical criteria, AF615 was selected as the most potent and specific inhibitor. Potency, specificity as well as mechanism of action of the selected compound was further investigated by applying biochemical and cell-based assays. Compound AF615 induced DNA damage, blockage of DNA synthesis and cell cycle arrest. Moreover, compound AF615 exhibited selectivity to cancer as opposed to normal cells. Reduced CDT1 or Geminin expression was sufficient to modulate DNA damage response and cell survival upon treatment with AF615. By applying an AlphaScreen™ HTS assay, we discovered a small molecule inhibitor of CDT1/Geminin protein complex, which facilitates effective targeting of cancer cells, thereby providing a promising lead compound that could serve as a chemical scaffold for further chemical optimization.

## Materials and Methods

### Cloning, Expression and Protein Purification

The bacterial expression plasmids for His-tagged human CDT1 variants, including CDT1^158−396^ (miniCDT1) and CDT1^158−356^ (tCDT1) have been described previously (De Marco et al., 2009). His-tagged human CDT1 mutants, including CDT1^158−396^Y170A (miniCDT1Y170A), CDT1^158−396^F173A (miniCDT1F173A) and CDT1^158−396^L176A (miniCDT1L176A) were cloned into vector pET28a (Novagen). Full-length human Geminin and Flag-tagged Geminin^28-209^ (ΔDBGeminin) were cloned into vector pET22b (Novagen). All constructs were generated by standard PCR-based techniques and all sequences were verified. To produce proteins either individually or as complexes, CDT1 and Geminin variants were expressed or co-expressed in Rosetta 2 (DE3) (Novagen). Cells were grown at 37°C and expression was induced with 0.1 mM IPTG for 2–3 h at 30°C. All proteins were purified by immobilized metal affinity chromatography (IMAC) using a Ni-NTA column, followed by size-exclusion chromatography (SEC). A Hi Load Superdex 200 column (GE Healthcare) and a Hi Load Superdex 75 column (GE Healthcare) were used in case of protein complexes and individual proteins respectively. Elution of proteins was carried out in Tris-HCl 25 mM (pH 7.5) and NaCl 200 mM.

Expression and purification of miniCDT1L176A individually was significantly restrained due to limited protein solubility and thus we were not able to isolate it in a form adequate for SPR analysis. All other proteins were essentially pure as verified by SDS-PAGE.

### Small-Molecule Compound Library

The library screened, belonging to the Netherlands Cancer Institute (NKI), consisted of 23,360 compounds arrayed in 384-well plates as single compounds at 10 mM in DMSO. All compounds were purchased from Specs company and their quality was assured by the vendor as greater than 90% pure. The library was screened at a constant of 1:2,000 dilution, with a 5 μΜ final concentration of compound in each well (0.4% DMSO).

### Surface Plasmon Resonance

Surface plasmon resonance spectroscopy (SPR) was performed at 25°C, using a Biosensor Biacore instrument (Biacore T100). About 6.000 RU of tCDT1 (residues 158–356) were immobilized on a CM5 Chip (Biacore) at pH 5.5, using amino coupling of Lysine residues. A series of concentrations of full-length Geminin and the compound AF615 were injected across the chip, reciprocally, in a buffer containing 20 mM Hepes pH 7.5, 200 mM NaCl, Tween 20 0.01% at a flow rate of 30 μl/min. The concentrations used were 12, 36, 107, 322, and 966 nM for Geminin and 10 μΜ, 100 μΜ, 1 mM for the compound AF615. The compound AF615 was used in the presence of 20% DMSO, to ensure high solubility. To study the interaction between full-length Geminin and miniCDT1 (residues 158–396) variants, about 5.000 RU of miniCDT1 wild type, miniCDT1Y170A and miniCDT1F173A were immobilized on a Ni NTA Chip (Biacore) via their N-terminal His-tag. Concentration series of full-length Geminin were injected across the chip, in a buffer containing 20 mM Tris pH 7.5, 200 mM NaCl, at a flow rate of 30 μl/min. Geminin was used at 1, 1.5, 2.5, 4, 6, 10, 15, 25, 40, 60, and 100 nM. Binding curves were recorded for each condition, using the empty flow cell as reference. All experiments were repeated at least two times in a non-sequential manner to exclude systematic errors. The Biacore T100 evaluation software was used for the initial data analysis.

### High-Throughput *In Vitro* Screening

High-throughput screening (HTS) of 23,360 synthetic compounds (Specs) at 5 μM final concentration in 0.4% DMSO in 384-well plates) was performed using the AlphaScreen™ Histidine (Nickel Chelate) Detection Kit (PerkinElmer). Final concentrations were 300 nM ΔDBGeminin/miniCDT1Y170A complex, 10 μg/ml beads (donor and acceptor) and 25 nM biotinylated anti-Flag antibody (Sigma) in 25 mM Hepes pH 7.4, 100 mM NaCl and 0.2% BSA in a final volume of 25 μl per well. Each plate was incubated for a minimum of 3 h in the dark at room temperature prior to measuring AlphaScreen™ signal (Envision reader, PerkinElmer). Initial hits were counter-screened for non-specific loss of His binding using AlphaScreen™ Biotinylated-HIS6 (PerkinElmer). Final concentrations were 20 nM Biotinylated-HIS6 peptide and 10 μg/ml beads (donor and acceptor), in the same buffer and final volume as above.

### AlphaScreen™ Assays

For the competition assay, an AlphaScreen™ Histidine (Nickel Chelate) Detection Kit, which includes streptavidin-coated Donor beads and nickel-chelated Acceptor beads, was purchased from PerkinElmer. Donor and Acceptor beads were brought together through the Flag-tagged Geminin/His6-tagged CDT1 interaction, in the presence of a biotinylated anti-Flag antibody (Sigma). The Nickel-chelated Acceptor beads as well as the Streptavidin Donor beads were used according to the manufacturer’s instructions. The optimum working concentration of the protein complex was determined at 50 nM. The constitution of the assay buffer was 25 mM Hepes pH 7.4, 100 mM NaCl and 0.1% BSA. The plate was incubated in the dark at RT for 1.5 h, before being measured in a PerkinElmer Envision plate reader.

To generate dose-response curves by applying AlphaScreen™ technology, nickel-chelated Acceptor beads and a-Flag Donor beads were brought together through the Flag-tagged ΔDBGeminin/His_6_-tagged miniCDT1 interaction. An 11-fold serial dilution with a starting concentration of 200 μM of compound AF615 was then used and the AlphaScreen™ signal was measured for each concentration.

### Cell Culture

MCF7 parental cells (ATCC) were maintained in Dulbecco’s modified Eagle’s medium (Invitrogen), supplemented with 20% fetal bovine serum (FBS; Invitrogen) and antibiotics, penicillin 100 U/ml and streptomycin 0.1 mg/ml (Invitrogen) at 37°C in a humid atmosphere with 5% CO_2_. U2OS (ATCC) and Saos-2 parental cells (from V. Gorgoulis), were cultured in Dulbecco’s modified Eagle’s medium, supplemented with 10% fetal bovine serum and antibiotics penicillin 100 U/ml and streptomycin 0.1 mg/ml. MCF10A cells (from C. Niehrs) were cultured in Dulbecco’s modified Eagle’s medium-F12 (Sigma) supplemented with 5% heat-inactivated horse serum (Biosera), insulin 10 μg/ml (Sigma), cholera toxin 100 ng/ml (Sigma), hydrocortisone 0.5 μg/ml (Sigma), human EGF 20 ng/ml (Sigma), penicillin 100 U/ml and streptomycin 0.1 mg/ml. hTERT-RPE1 cells (from M. Bettencourt-Dias) were maintained in Dulbecco’s modified Eagle’s medium-F12, supplemented with 10% fetal bovine serum and antibiotics. MCF7 CDT1-GFP (Xouri et al., 2007) and MCF7 GFP-NLS (Symeonidou et al., 2013) were maintained in Dulbecco’s modified Eagle’s medium supplemented with 20% fetal bovine serum, antibiotics penicillin/streptomycin and 500 μg/ml geneticin (Gibco). Cells were routinely subjected to *mycoplasma* testing and found to be negative.

### FRET Measurements and Analysis

FRET was determined by the sensitized emission method. For sensitized emission FRET, MCF7 CDT1-GFP stable cells were seeded on CELLview™ Cell Culture Slides (Greiner Bio One) (7,500 cells/well) and after 24 h they were transfected with 1 μg of Geminin-dHcRed expressing vector with Polyethylenimine transfection reagent (Polysciences). MCF7 GFP-NLS cells transiently transfected with Geminin-dHcRed were used as negative control. After 24 h incubation with different compound AF615 concentrations, cells were fixed for 15 min at room temperature with 4% paraformaldehyde. Nuclei were counterstained with Hoechst (Invitrogen). Sensitized emission FRET images were acquired in an automated fashion with a ScanR inverted microscope High-content Screening Station (Olympus), equipped with a 20X dry objective, using the ScanR acquisition software. % FRET efficiency was estimated with a custom ImageJ/Fiji macro. At least 300 cells were analysed per condition.

### Immunofluorescence

For experiments in multiwell imaging plates: Cells were seeded on 384 well plates (Greiner Bio-One) using a microplate dispenser (Mettler-Toledo) at a density of 1,500 cells/well, respectively. Following any indicated treatment, cells where fixed in 4% paraformaldehyde for 15 min at room-temperature and permeabilized in PBS containing 0.5% Triton X-100 for 15 min using a Liquidator (Mettler-Toledo). When Click-iT EdU staining was performed, cells were incubated with 10 μM EdU 30 min before fixation and EdU detection was performed according to the manufacturer’s recommendations (Thermo Scientific) before incubation with primary antibodies. Cells were incubated with primary antibodies diluted in PBS-Triton X-100 0.1% for 1 h at room temperature. Primary antibodies used: 53BP1 (rabbit, 1–1000, Abcam), Phospho-Histone H2A.X-Ser139 (mouse, 1–1000, Merck Millipore), CDT1 (rabbit, 1–500, custom antibody produced in house), Geminin (rabbit, 1–1000, custom antibody produced in house), CyclinA (mouse, 1–500, Neomarkers). Multiwell plates were washed three times with PBS 1X. Following primary antibody incubation, cells were incubated for 1 h with secondary antibodies conjugated with Alexa Fluor 488 (1–1000, Invitrogen), Alexa Fluor 568 (1–1000, Invitrogen), Alexa Fluor 647 (1–1000, Invitrogen). Multiwell plates were washed three times with PBS 1X. Cell nuclei were counterstained with Hoechst 33342 (Invitrogen). After being washed three more times in PBS 1X, plates were subjected to image acquisition. For immunofluorescence on coverslips: Cells were grown on glass 13-mm-wide, 1.5-mm-thick autoclaved glass poly-D-lysine (Sigma) coated coverslips and following treatment cells were fixed in 4% paraformaldehyde for 15 min at room-temperature and permeabilized in PBS containing 0.5% Triton X-100 for 5 min. Cells were blocked with PBS containing 10% FBS, 3% BSA for 1 h and then were incubated with primary antibodies overnight at 4°C. Following primary antibody incubation, coverslips were washed three times with PBS containing 0.1% Tween and then cells were incubated for 1 h with secondary antibodies conjugated with Alexa Fluor 488 (1–1000, Invitrogen) and Alexa Fluor 568 (1–1000, Invitrogen). Cell nuclei were counterstained with Hoechst 33342 (Invitrogen) and after being washed three times with PBS containing 0.1% Tween, coverslips were washed in distilled water, dried on paper, and mounted in Mowiol-based mounting medium (Calbiochem). For micronuclei and anaphase bridges detection, cells were stained following indicated treatment with Hoechst 33342 (Invitrogen) and micronuclei and anaphase bridges were scored with ImageJ/FIJI analysis software. At least 500 nuclei were counted for micronuclei quantification and at least 100 anaphases were counted for anaphase bridges.

### High Content Imaging

Images were acquired with a ScanR inverted microscope High-content Screening Station (Olympus) equipped with wide-field optics, a 10X or 20X UPSALO dry objective, fast excitation and emission filter-wheel devices for DAPI, FITC, Cy3, and Cy5 wavelengths. Images were acquired in an automated manner with the ScanR acquisition software (Olympus, 2.5.0). Depending on cell numbers, five images were acquired containing at least 3,000 cells per well. Images were processed and analysed with an automated way with KNIME platform analytics software (Version 3.7.0) using a custom-built pipeline. A median filter and automated threshold were applied to nuclei images and then automated nuclei segmentation was performed. Α dynamic background correction was applied to all images. The nuclei mask was then applied to analyze pixel intensities in different channels for each individual nucleus. CSV files containing all the data from the automated analysis were processed with Excel and plotted using Graph Pad Prism 6.

### Crystal Violet Sensitivity Assays

Cells were seeded at 12.5 × 10^4^ cells per well into 24-well plates in three replicates per condition, and 24 h after plating were treated with the appropriate compound concentrations for 3 days, with daily medium and drug replacement. Surviving cells were fixed and stained with crystal violet (Sigma). For siRNA experiments, cells were seeded at 2 × 10^4^ per well into 24-well plates, and 24 h after plating were transfected with siRNAs. After 24 h cells were treated with the appropriate compound concentrations for 24 h and then surviving cells were fixed and stained with crystal violet.

### Flow Cytometry

All cell lines were harvested by trypsinization and fixed using ice-cold 70% ethanol (added drop wise) and stored at −20°C overnight. Then cells were centrifuged at 2,000 g for 15 min at 4°C, rinsed twice in 1X PBS and then stained with 2 mg/ml propidium iodide (Sigma)/100 mg/ml RNase A (Sigma) dissolved in 1X PBS for 30 min. Cellular DNA content was assessed on a FACS Calibur flow cytometer using the CellQuest Pro software (BD Biosciences) and analyzed with Flowjo (BD Biosciences).

### Plasmid DNA, siRNA Transfections and Drug Treatments

For plasmid transfections, cells were transfected at 70–90% confluency using Polyethylenimine transfection reagent (Polysciences) or Lipofectamine 2000 transfection reagent (Invitrogen) according to manufacturer’s instructions. Plasmids used: CDT1-GFP (cloned in pCDNA 3.1/EGFP), Geminin-dHcRed (pdiHcRed-N1), GFP (pCDNA3.1/EGFP) and dHcRed (pdiHcRed-N1) were used from Xouri et al. (2007). Transfections of siRNA duplexes were performed with Lipofectamine RNAiMAX (Invitrogen) according to manufactures instructions. siRNAs used: siLuciferase: 5′ CGUACGCGGAAUACUUCGAdTdT 3′, siCDT1 #1: 5′ AACGUGGAUGAAGUACCCGACdTdT 3′, siCDT1 #2: 5′ CCUACGUCAAGCUGGAdTdT 3′ were used in final concentration of 100 nM for 48 h and were purchased from Eurofins MWG. Pre-designed siGeminin: 5′ GAAUGACCACUUAACAUCUdTdT 3′ and Nontargeting siRNA were purchased from Thermo Scientific (Ambion negative control #1) and were used in a final concentration of 20 nM for 24 h of incubation. The following reagents were used to treat cells for the indicated time at the indicated final concentration before collection: HU (Hydroxyurea, Sigma), Etoposide (Sigma), CPT (Camptothecin, Sigma).

### Statistical Analysis

Data are expressed as the mean ± SD for *n* = 3 biological independent replicates. The two-sample Student *t* test was employed to compare the means between groups and all of the data were considered to be significant at *p < 0.05, **p < 0.01, ***p < 0.001, ****p < 0.0001. For mean intensity quantifications statistical significance was determined using Mann–Whitney *U*-tests. Statistical analysis was performed using GraphPad Prism 6 (GraphPad Software).

## Results

### ΔDBGeminin/miniCDT1^Y170A^ Mutant Complex Constitutes an Appropriate Target Molecule for Developing an HTS Assay to Discover Chemical Compounds That Inhibit the Geminin-CDT1 Protein Complex Formation

Geminin-CDT1 protein interaction displays very high binding affinity (K_D_ = 4.4 ± 2.2 nM) (Lee et al., 2004; De Marco et al., 2009), representing a stable protein complex. For this reason, a Geminin/CDT1 mutant complex exhibiting lower binding affinity than the wild type variant, would make the identification of small molecule inhibitors more favourable. Therefore, we have examined whether single point mutations of critical CDT1 residues could weaken the high affinity of Geminin-CDT1 interaction. It has been previously described that mouse Cdt1 domain 172–368 a.a. is the minimal Cdt1 fragment that interacts with Geminin (Lee et al., 2004). This fragment is conserved among the species and corresponds to human CDT1 158–396 a.a. region (herein named miniCDT1). Moreover, crystallographic analysis (Lee et al., 2004) has shown that mouse Cdt1 residues Y183, F186 and L189 (corresponding to the human CDT1 Y170, F173 and L176 residues, respectively) are crucial for the protein complex formation. Therefore, these highly conserved Geminin-contacting residues of CDT1 were mutated to alanines and their effect on the stability of the Geminin-CDT1 protein complex was assessed.

Initially three single mutants Y170A, F173A and L176A were introduced into the miniCDT1 protein sequence (Supplementary Figures S1A–H). All three miniCDT1 mutant variants were bacterially co-expressed and subsequently co-purified with a truncated version of human Geminin (residues 28–209, ΔDBGeminin) that included the full amino-acid sequence of Geminin, apart from the first 28 residues flanking the destruction box signature of the protein. To examine whether the three single CDT1 mutations (Y170A, F173A and L176A) could lower the binding affinity of CDT1 to Geminin and thus contribute to a more easily disruptable complex than the wild type variant, we performed a competition assay by applying AlphaScreen™ technology (Supplementary Figure S2A). We examined whether increasing concentrations of the untagged ΔDBGeminin could displace the Flag-tagged ΔDBGeminin and thus reduce AlphaScreen™ signal, indicating disruption of ΔDBGeminin/miniCDT1 complex. Using an equimolar amount of untagged ΔDBGeminin led to nearly complete loss of AlphaScreen™ signal (∼92% reduction) in the case of CDT1^Y170A^ mutant. For CDT1 mutations F173A and L176A, a 10-fold and 100-fold excess amount was needed respectively, to achieve a similar signal reduction (Supplementary Figure S2A). These data indicate that CDT1^Y170A^ mutant has the most pronounced effect on disruption of the protein complex compared to CDT1^F173A^ and CDT1^L176A^ mutants.

**FIGURE 1:**
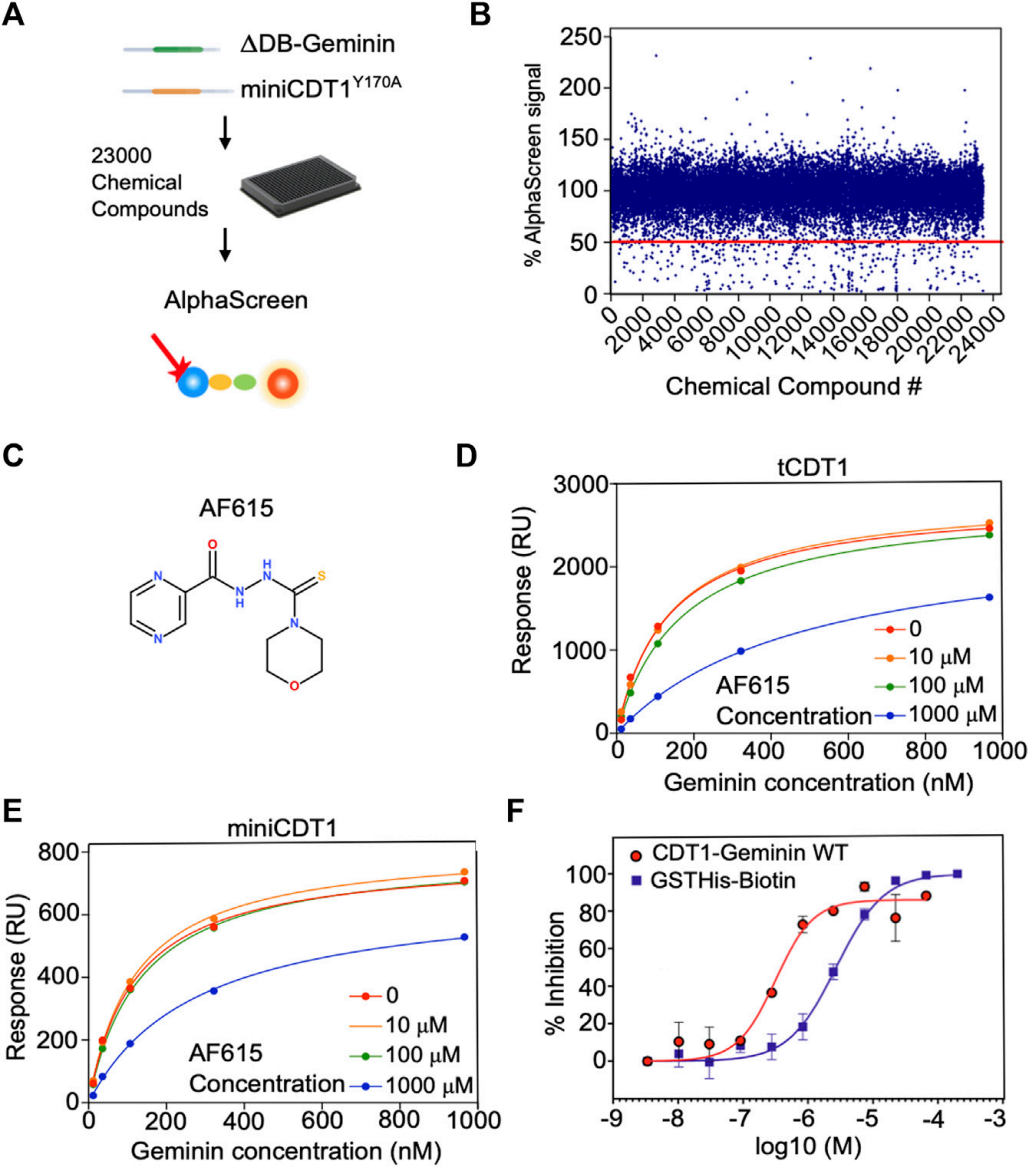
Chemical compound AF615 disrupts the CDT1-Geminin protein complex *in vitro*. **(A)** Schematic representation of the high-throughput screening for the identification of chemical compounds inhibiting the CDT1-Geminin protein interaction complex using AlphaScreen technology. ΔDB-Geminin and miniCDT1Y170A mutant proteins were used as screening targets. **(B)** *In-vitro* high-throughput screening for chemical compounds inhibiting the CDT1-Geminin protein-protein interaction. In total 23,360 compounds were screened at a single concentration of 5 μM using a 384-well plate format. Using a selection criterion of >50% signal reduction (solid line), 360 primary “hits” were identified and subjected to further validation. The x-axis represents the different compounds, while the y-axis represents the percentage (%) of AlphaScreen signal, as normalized against the median value of AlphaScreen signal in presence of the compounds arrayed in each plate. **(C)** Chemical structure of the chemical compound AF615. **(D,E)** Compound AF615 inhibits Geminin binding to CDT1 in a dose dependent manner. Graphical representation of Surface Plasmon Resonance (SPR) analysis of tCDT1 and miniCDT1 binding to Geminin in the presence of different concentrations of compound AF615. The x-axis represents Geminin concentrations, while the y-axis represents the Response (RU). **(F)** Inhibition of CDT1-Geminin wild type complex formation in a dose dependent manner in the presence of AF615 using Alphascreen technology. X-axis represents compound concentration on a logarithmic scale, while y-axis represents percentage (%) inhibition. AlphaScreen Signal was determined for each compound concentration in presence of the Geminin/CDT1 wild type complex (red line) and a single fusion protein containing both a GST- and a His-tag (blue line), as a control. Each data point is the mean ± SD from duplicate measurements. Data points were fitted using the non-linear regression function log (inhibitor) versus response variable slope (four parameters).

To biochemically characterize the impact of the above three single CDT1 mutations on the stability of the protein complex, we determined the dissociation constant (K_D_), by utilising surface plasmon resonance. Each of the CDT1 variants (wild type, Y170A and F173A mutants) assessed by the AlphaScreen™-based competition assay was purified following bacterial expression and immobilized to a Ni NTA Biacore chip via an N-terminal His-tag. Subsequently, a wide range of increasing full length Geminin concentrations (0, 1, 1.5, 2.5, 4, 6, 10, 15, 25, 40, 60, and 100 nM) was injected over the chip (Supplementary Figures S2B–D). Purification of miniCDT1^L176A^ mutant could not meet the standards required for an adequate SPR analysis due to limited protein solubility and thus no SPR data could be obtained. Geminin bound to all three immobilized miniCDT1 variants dose-dependently. Our data analysis revealed a dissociation constant (K_D_) of 9.4 nM for the binding of Geminin to miniCDT1 wild type. ΜiniCDT1^Y170A^ mutant exhibited a binding affinity reduced by a factor of four (K_D_ = 41 nM), while miniCDT1^F173A^ mutant showed an intermediate binding affinity (K_D_ = 25 nM). The calculated K_D_ values agreed with the data obtained through our AlphaScreen™-based competition assay (Supplementary Figures S2B–D) and suggest that ΔDBGeminin/miniCDT1^Y170A^ mutant complex facilitates the detection of Geminin-CDT1 protein interaction *in vitro* and can also provide a protein complex easy to disrupt, thus favouring the identification of small molecule inhibitors. Based on the above, we decided to select ΔDBGeminin/miniCDT1^Y170A^ as our screening target molecule (Figure 1A).

### Development and Optimisation of AlphaScreen™ Assay

To identify inhibitors able to disrupt the Geminin-CDT1 protein complex, we developed a high-throughput screening assay based on the Amplified Luminescence Proximity Homogeneous Assay (AlphaScreen™) technology. To this end, an AlphaScreen™ Histidine (Nickel Chelate) Detection Kit, which includes streptavidin-coated Donor beads and nickel-chelated Acceptor beads was used. Beads were brought in proximity through the Flag-tagged ΔDBGeminin/His_6_-tagged miniCDT1^Y170A^ interaction in the presence of a biotinylated anti-Flag antibody.

To find the optimal assay conditions ensuring compatibility with high-throughput format as well as adequate signal-to-background ratio, the appropriate concentration for Geminin/CDT1 protein complex and AlphaScreen™ beads was determined. To achieve this, Geminin/CDT1 and beads were titrated separately over a wide range of concentrations and AlphaScreen™ signal was measured. A concentration range of 200–400 nM and a final concentration of 10 μg/ml for Geminin/CDT1 complex and beads respectively, led to a greater than 20-fold signal-to-background ratio (data not shown). The biotinylated anti-Flag antibody was constantly used at 25 nM according to the manufacturer’s instructions. The above-described optimized conditions were finally selected for application of our HTS assay.

To assess validity and robustness of our AlphaScreen™ HTS, Z′ factors were extracted for each screening plate (Supplementary Figure S2E). With an average Z′ value of 0.6, the assay performance and robustness were considered as adequate.

The high-throughput screening was carried out using a 384-well plate format. Out of 23,000 small molecules initially screened, 310 reduced the AlphaScreen™ signal by >50% (Figures 1A,B). All 310 hits were subsequently subjected to a counter-screen to exclude those that no longer resulted in the same percentage of signal reduction (>50%), implying nonspecific target-independent activity due to possible interference with the format of our primary screening. In the counter-screen, a Biotin-His_6_ peptide was used to bring the streptavidin-coated Donor beads and nickel-chelated Acceptor beads in close proximity. The 310 compound hits were then ranked based on the ratio of signal reduction (%) observed in presence of the Biotin-His_6_ peptide and the Flag-tagged ΔDBGeminin/His_6_-tagged miniCDT1^Y170A^ protein complex. From the 310 validated hits, compound AF615 was selected as the most potent and selective inhibitor of Geminin-CDT1 protein interaction (Figure 1C) and subjected to further experimental analysis.

### Selected Compound AF615 Inhibits Geminin/CDT1 Complex Formation *In Vitro*, in a Dose-Dependent Manner

To determine the affinity of Geminin-CDT1 interaction in the presence of compound AF615 and thus provide further insights in terms of potency, we performed surface plasmon resonance analysis (Figures 1D,E). To achieve this, miniCDT1 (residues 158–396) was immobilized covalently to a CM5 Biacore chip. Moreover, to enhance the detection of the compounds’ inhibitory effect, a shorter CDT1 protein fragment named tCDT1 (residues 158–356) was additionally utilized. Both CDT1 protein variants display equivalent protein complex forming ability (Lee et al., 2004; De Marco et al., 2009). Following miniCDT1 and tCDT1 immobilization compound AF615 was injected across the chip in a range of increasing concentrations (10 μΜ, 100 μΜ, 1 mM) together with full length Geminin. Our data analysis revealed an inhibition of Geminin binding to CDT1 upon treatment with compound AF615 (1 mM) (Figures 1D,E). More specifically, AF615 displayed a KI = 0.37 μM in case of Geminin-tCDT1 interaction (Figure 1D) and a KI = 0.75 μM in case of Geminin-miniCDT1 interaction (Figure 1E). To further quantify the inhibitory effect of AF615, we generated dose-response curves by applying AlphaScreen™ technology. Nonlinear regression analysis revealed a potency in the low micromolar range for AF615 (IC_50_ = 0.313 μM) regarding its ability to inhibit Geminin/CDT1 complex formation *in-vitro* (Figure 1F). The IC_50_ value of compound AF615 is also in agreement with the obtained KI values through our SPR analysis.

As a control of specificity, we used a single fusion protein containing both a GST- and a His-tag, where we observed an inhibitory effect using an IC50 = 2.8 μΜ (about one order of magnitude higher than our protein complex). The structural similarity of compound AF615 with imidazole (known for competing with His-tag) could account for the mild inhibitory effect observed in the control condition.

### Compound AF615 Inhibits CDT1/Geminin Interaction in Cancer Cells

**FIGURE 2:**
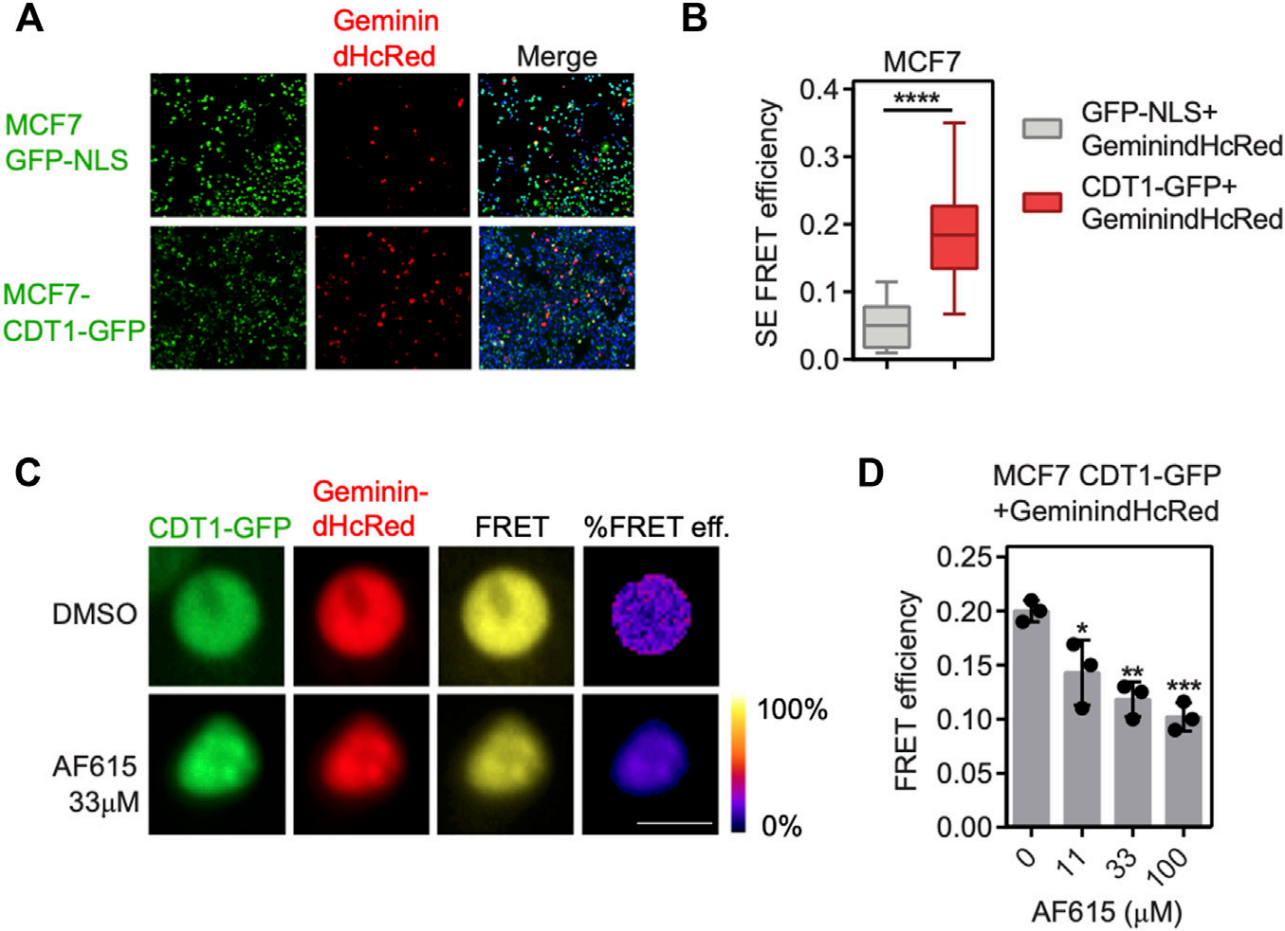
Compound AF615 inhibits CDT1-Geminin protein-protein interaction in cancer cells. **(A)** Representative images of MCF7CDT1-GFP and MCF7GFP-NLS transiently transfected with Geminin-dHcRed for 24 h. **(B)** Graphical representation of the quantification of SE-FRET efficiency. **(C)** Representative images of SE-FRET of MCF7CDT1-GFP transfected with Geminin-dHcRed treated for 24 h with 33 μΜ compound AF615. GFP-NLS + Geminin-dHcRed vs. CDT1-GFP + Geminin-dHcRed, ****p < 0.0001. **(D)** Quantification of the SE-FRET efficiency. Dots represent the mean FRET values of three independent biological repetitions. 0 μΜ AF615 vs. 11 μM AF615, *p < 0.05; 0 μΜ AF615 vs. 33 μM AF615, **p < 0.01; 0 μΜ AF615 vs. 100 μM AF615 ***p < 0.001. Statistical analysis was performed using two-tailed Student’s *t*-tests. Scale bars: 7 μm.

To provide further evidence for the ability of the compound AF615 to inhibit the formation of the Geminin/CDT1 complex in mammalian cells, we performed quantitative analysis of Geminin/CDT1 interaction using FRET (Förster resonance energy transfer). FRET efficiency was measured between CDT1-GFP and Geminin-dHcRed, using the sensitized emission method. Asynchronous MCF7 cells stably expressing CDT1-GFP were transiently transfected with Geminin-dHcRed, and after 24 h sensitized emission FRET was performed (Figure 2A). MCF7 cells stably expressing GFP-NLS, transiently transfected with Geminin-dHcRed were used as negative control. As shown in Figure 2B, an almost three times higher FRET mean intensity was observed in cells expressing simultaneously CDT1-GFP and Geminin-dHcRed, compared to cells expressing GFP-NLS and Geminin-dHcRed, indicating interaction of CDT1 and Geminin. The interaction of CDT1 with its inhibitor Geminin in living cells, has also been previously evaluated using fluorescence lifetime imaging microscopy (FLIM) (Xouri et al., 2007). To examine the ability of compound AF615 to inhibit the interaction of CDT1 and Geminin in mammalian cells, we repeated the sensitized emission FRET experiments in the presence of different concentrations of AF615. In more detail, asynchronous MCF7 CDT1-GFP stable cells were transiently transfected with Geminin-dHcRed and after 24 h cells were treated with three different concentrations of AF615 (11, 33, and 100 μΜ) or DMSO for 24 h. As shown in Figures 2C,D treatment of MCF7 cells with 33 and 100 μΜ of compound AF615 reduced the FRET mean intensity of CDT1-GFP and Geminin-dHcRed interaction almost 50%, compared to DMSO treated cells. To verify that the inhibition of the CDT1-Geminin interaction by compound AF615 is specific, we repeated the experiment using the MCF7-GFP NLS cells. We did not observe any FRET signal in MCF7-GFP NLS cells expressing Geminin-dHcRed treated with increasing concentrations of compound AF615 (11, 33, and 100 μΜ) (Supplementary Figure S2F). Moreover, treatment of compound AF615 did not affect the levels of exogenously expressed Geminin-dHcRed, supporting the idea that the inhibition of CDT1-Geminin interaction by compound AF615 is specific (Supplementary Figure S2G).

### Compound AF615 Activates the DNA Damage Response and Blocks DNA Synthesis in Cancer Cell Lines

**FIGURE 3:**
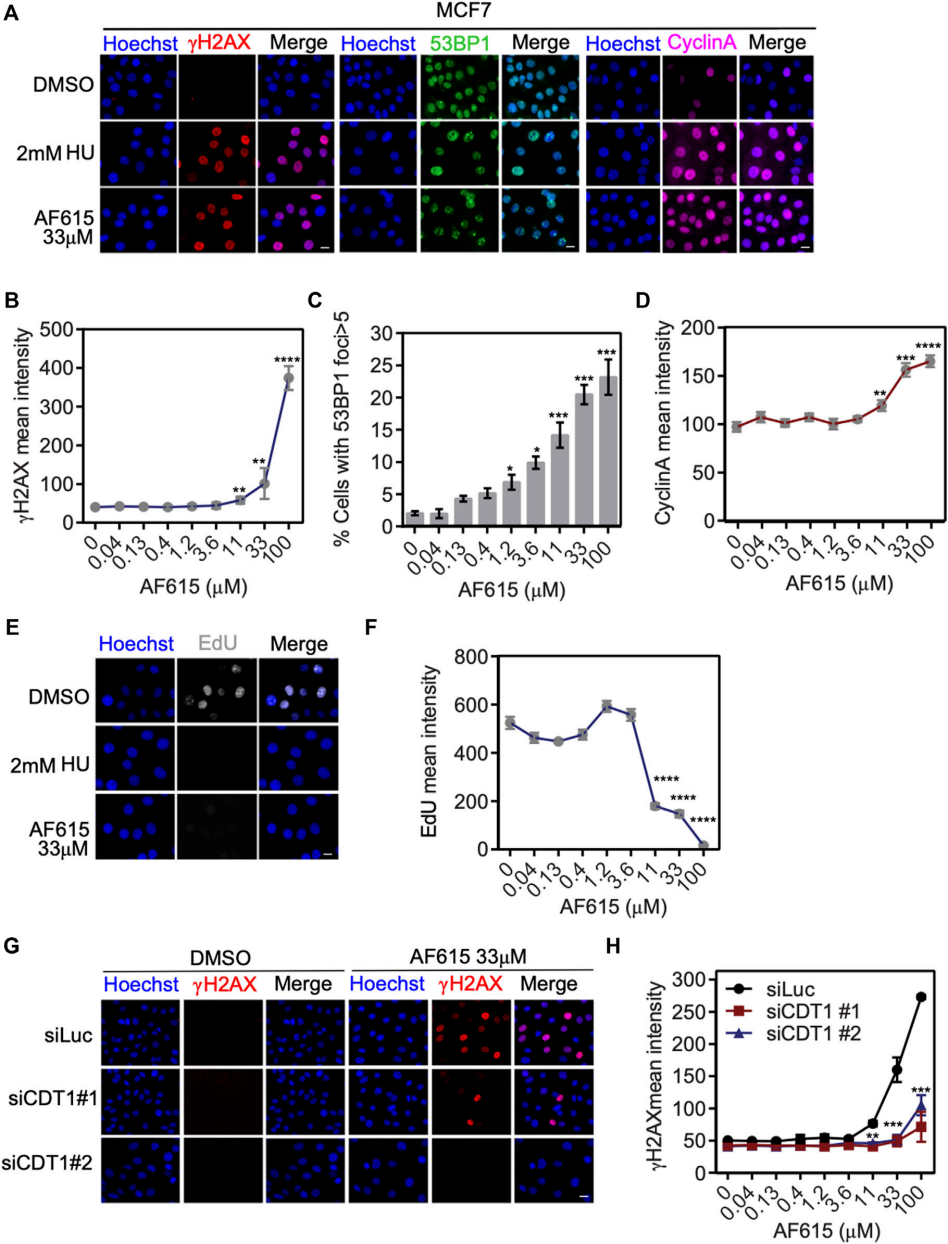
Compound AF615 induces DNA damage and blocks DNA synthesis in cancer cells. **(A)** Representative images of MCF7 cells treated with 33 μΜ compound AF615 for 24 h and immunostained for γH2ΑΧ, 53BP1 and CyclinA. Nuclei were counterstained with Hoechst. Control, 2 mM HU (Hydroxyurea) for 24 h. **(B)** Quantitative analysis of γH2ΑΧ mean intensity using high-content imaging and high-throughput automated image analysis. Graph depicts the mean γH2ΑΧ intensity of *n* = 3 biological independent experiments per compound AF615 concentration. **(C)** Quantification of the percentage of cells with 53BP1 foci (>5 foci per nucleus) per compound AF615 concentration. Graph depicts the mean % percentage of cells with 53BP1 foci of *n* = 3 biological independent experiments per compound AF615 concentration. **(D)** Quantitative analysis of CyclinA mean intensity using high-content imaging and high-throughput automated image analysis. Graph depicts the mean CyclinA intensity of *n* = 3 biological independent experiments per compound AF615 concentration. **(E)** Representative images of the control, HU (2 mM) and compound AF615 (33 μΜ) treated MCF7 cells labeled with EdU. Nuclei stained with Hoechst. **(F)** Quantitative analysis of EdU intensity per compound AF615 concentration. Graph depicts the mean EdU intensity of *n* = 3 biological independent experiments per compound AF615 concentration. **(G)** Representative images of MCF7 transfected with siRNA for CDT1, treated with 33 μΜ compound AF615 and immunostained for γH2ΑΧ. MCF7 cells transfected with siLuciferase were used as control. **(H)** Graph represents the quantification of γH2ΑΧ mean intensity from *n* = 3 biological independent experiments. For all conditions and replicates, at least 2,000 nuclei were analyzed. ****p < 0.0001, ***p < 0.001, **p < 0.01, *p < 0.05. Statistical analysis determined with two-tailed Student’s *t*-tests. Scale bars: 7 μm.

We have provided evidence that treatment with compound AF615 leads to reduced interaction of CDT1 and Geminin both *in-vitro* and in cancer cells. Previous studies have shown that, overexpression of CDT1, or knock-down of Geminin, leads to accumulation of double strand breaks, cell cycle arrest and apoptosis in cancer cell lines. In order to examine whether treatment with compound AF615 has a similar phenotype, we examined the activation of DNA damage response, the progression of the cell cycle and DNA synthesis in cancer cell lines in the presence of AF615. MCF7 breast adenocarcinoma cells were treated with increasing concentrations of the compound AF615 (0.04, 0.13, 0.4, 1.2, 3.6, 11, 33, and 100 μΜ) and after 24 h, cells were immunostained for γΗ2ΑΧ and 53BP1. High content imaging analysis in MCF7 cells treated with 11 μΜ and higher concentrations of compound AF615, revealed increased Η2ΑΧ phosphorylation (Figures 3A,B) and 53BP1 foci formation (Figures 3A,C). Moreover, in the presence of 11 μΜ and higher concentrations of compound AF615, the fraction of cells expressing CyclinA, which indicates cells undergoing S and G2 phase, was increased compared to DMSO treated cells (Figures 3A,D). In order to monitor DNA synthesis, MCF7 cells were cultured in the presence of different concentration of compound AF615 and pulsed-labeled with EdU for 1 h and then subjected to high-content imaging (Figure 3E). The EdU signal was reduced almost three times in cells treated with 11 μΜ or higher concentrations of the compound AF615 (Figure 3F), in agreement to the accumulation of DNA damage. In accordance with the data obtained from MCF7 cells, increased phosphorylation of H2AX (Supplementary Figures S3A,B), blockage of DNA replication indicated by reduced EdU incorporation was also evident in U2OS and Saos-2 osteosarcoma cell lines treated with 11 μM and higher concentrations of compound AF615 (Supplementary Figures S3C,D). In order to examine whether activation of the DNA damage response occurring upon compound AF615 treatment was linked to CDT1 activity, we performed knock-down experiments using siRNAs targeting CDT1 in MCF7 cells (Supplementary Figures S4A,B) and subsequently examined the activation of γH2AX (Figure 3G). Cells transfected with two different siRNAs for CDT1, were subjected to immunofluorescent experiments using anti-γH2AΧ antibody and the intensity of the staining was quantified. Cells treated with siCDT1 exhibited 3 times less phosphorylation of H2AX in the presence of 33 and 100 μΜ compound AF615, compared to control cells treated with siLuciferase (Figure 3H), suggesting that the induction of DNA damage mediated by the compound AF615 is partially dependent on CDT1. To verify that the rescue of DNA damage in cells treated with siCDT1 and compound AF615 is due to the lack of CDT1 and not to a reduction in the fraction of cells in S phase, we examined the incorporation of EdU in MCF7 cells transfected with siCDT1. Analysis revealed that siRNA-mediated knockdown of CDT1 in MCF7 cells did not affect the incorporation of EdU, suggesting that the reduction of the DNA damage in MCF7 cells treated with siCDT1 and compound AF615 is specific (Supplementary Figure S4C) This is further supported by the fact that depletion of CDT1 by siRNA did not affect the phosphorylation of H2AX in MCF7 cells treated with 2 mM HU, or 2 μΜ Etoposide or 2 μΜ Camptothecin, in contrast to compound AF615 treatment (Supplementary Figure S4D). Taken together, our results suggest that compound AF615 induces DNA damage and blocks DNA synthesis in cancer cell lines in a CDT1-dependent manner.

### Compound AF615 Blocks Cell Cycle Progression and Reduces Cell Viability of Different Cancer Cell Lines

**FIGURE 4:**
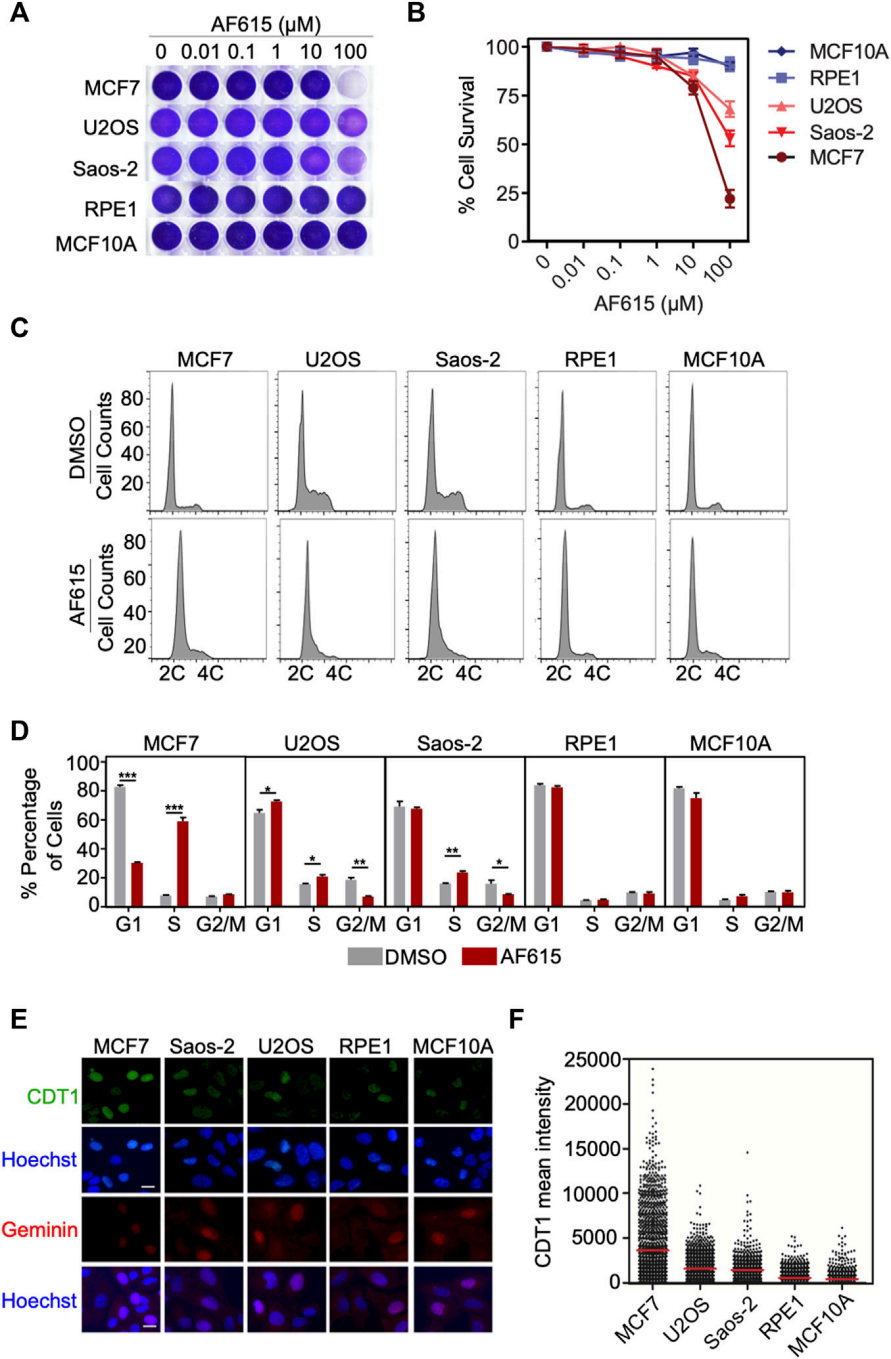
Cancer cells exhibit increased sensitivity to chemical compound AF615. **(A)** Survival assay for MCF7, U2OS, Saos-2, MCF10A, RPE1 cells treated with increasing concentrations of compound AF615 for 3 days. Cells stained with crystal violet. **(B)** Quantification of cell survival from *n* = 3 biologically independent experiments. **(C)** Flow cytometry (FACs) profiles of MCF7, U2OS, Saos-2, MCF10A and RPE1 cells treated with compound AF615 (100 μM) for 24 h. DNA content analysis was performed using propidium iodide (PI). Representative flow cytometry profiles of three biologically independent experiments are shown. 2C and 4C, DNA content of G1 and G2 cells, respectively. **(D)** Means and SDs (error bars) from *n* = 3 biologically independent experiments are shown, ***p < 0.001, **p < 0.01, *p < 0.05. Statistical analysis determined with two-tailed Student’s *t*-tests. **(E)** Representative images of MCF7, U2OS, Saos-2, MCF10A, RPE1 immunostained for CDT1 and Geminin. Nuclei counterstained with Hoechst. **(F)** Quantification of CDT1 mean intensity. Scale bars: 7 μm.

Overexpression of CDT1 or down–regulation of Geminin leads to DNA re-replication, double strand breaks and apoptosis in different cancer cell lines (Vaziri et al., 2003; Zhu and DePamphilis, 2009). Since compound AF615 phenocopies the cellular effects induced by abnormal CDT1 and/or Geminin expression, we examined whether it affects the proliferation of cancer cells. To this end we addressed the sensitivity of different cancer (MCF7, U2OS, Saos2) and normal cell lines (MCF10A, RPE1) to increasing concentrations of compound AF615 (0.01–100 μΜ) (Figure 4A). Treatment with 100 μΜ AF615 for 24 h resulted in a 75% reduction of MCF7 cell survival and a 50% reduction in the survival of U2OS and Saos-2 cells, respectively. We could not detect any change in the viability of RPE1 and MCF10A cells within the tested concentration of AF615 up to 100 μΜ (Figure 4B). CDT1 overexpression or Geminin depletion induces DNA re-replication and cell cycle arrest in S and G2 phase, especially in cancer cell lines (Vaziri et al., 2003; Melixetian et al., 2004; Tatsumi et al., 2006; Zhu and DePamphilis, 2009). To examine the cell cycle progression upon treatment with compound AF615, we performed FACS analysis in MCF7, U2OS, Saos-2, RPE1 and MCF10A cells. The different cell lines were treated with 100 μΜ compound AF615 for 24 h, stained with propidium iodide (PI) and DNA content was analyzed using flow cytometry. A five-fold increase in the percentage of cells undergoing S phase was evident in MCF7 cells following treatment with 33 and 100 μΜ of compound AF615 (Figures 4C,D; Supplementary Figure S5A). An increase in the percentage of S phase cells was also evident in the case of U2OS and Saos-2 cells, showing the same trend as MCF7 cells but not to the same extent (Figures 4C,D). On the contrary, the cell cycle progression of normal RPE1 and MCF10A cells treated with AF165 was not severely affected (Figures 4C,D). Since compound AF615 affected differently the cell cycle progression of cancer and normal cells, we hypothesized that this phenotype may be related to the expression levels of CDT1 and Geminin. Quantification of the endogenous expression levels of CDT1 in MCF7, U2OS, Saos-2, RPE1 and MCF10A cells revealed almost three times higher protein levels of CDT1 in MCF7, U2OS, and Saos-2 cells compared to RPE1 and MCF10A cell (Figures 4E,F). It has been previously shown that the ratio of CDT1/Geminin expression is critical for the induction of DNA re-replication. We quantified the ratio of CDT1 and Geminin protein levels in MCF7, U2OS, Saos-2, RPE1 and MCF10A and identified that MCF7, U2OS and Saos-2 exhibit abnormal ratios of CDT1 and Geminin expression (eight times higher expression of CDT1 compared to Geminin in MCF7 cells and three higher expression in U2OS and Saos-2 cells). On the contrary, RPE1 and MCF10A exhibited normal ratios of CDT1 compared to Geminin (Supplementary Figures S5B,C). The alterations in the expression levels of CDT1 and Geminin may explain the difference in cell cycle progression and viability upon compound AF615 treatment in the cell lines examined. Overall, these data suggest that cancer cells show increased sensitivity to compound AF615 in terms of cell proliferation and viability.

### Knock-Down of Geminin Enhances the Potency of Compound AF615 in Human Normal Cells

**FIGURE 5:**
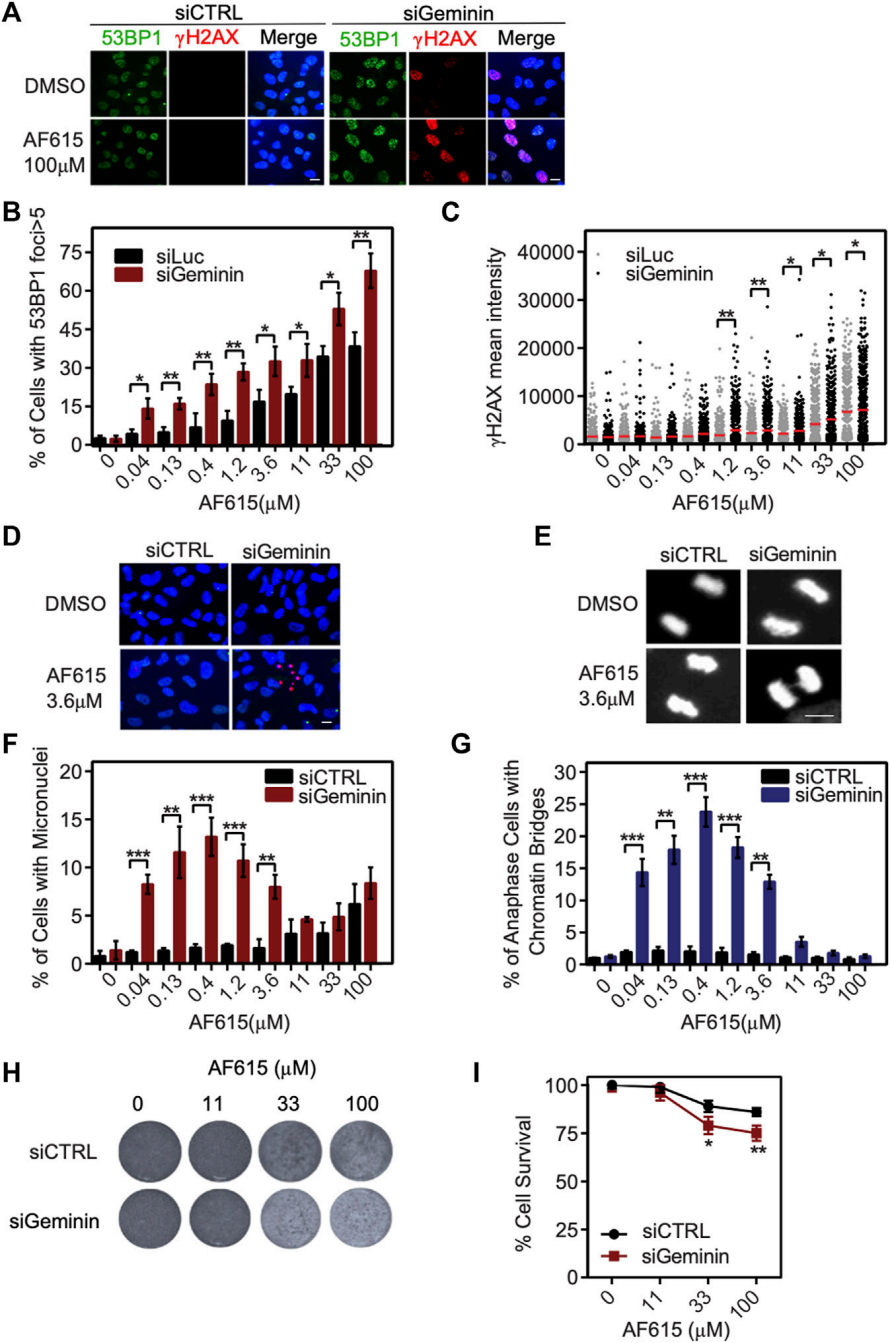
Geminin depletion enhances the potency of chemical compound AF615. **(A)** Representative images of RPE1 cells transfected with Geminin siRNA, treated with 33 μΜ compound AF615 for 24 h and immunostained for γH2ΑΧ and 53BP1. Nuclei were counterstained with Hoechst. RPE1 cells transfected with siCTRL were used as control. **(B)** Quantification of the % percentage of cells with 53BP1 foci. Statistical significance determined by two-tailed Student’s *t*-tests; **p < 0.01; *p < 0.05; *n* = 3 biologically independent replications. **(C)** Scatter plot depicts the per cell γH2ΑΧ mean intensity for the different compound AF615 concentrations examined. At least 1,000 cells were analyzed per condition and per experimental replicate. Statistical significance determined with Mann–Whitney *U*-tests; **p < 0.01; *p < 0.05; *n* = 3 biologically independent replications. **(D)** Representative images of micronuclei detected in RPE1 cells transfected with Geminin siRNA and treated with compound AF615 for 24 h. **(E)** Representative images showing chromatin bridges in anaphase cells following depletion of Geminin and treatment with compound AF615. **(F)** Graphical representation of the % percentage of cells harboring micronuclei. Means and SDs (error bars) from *n* = 3 biologically independent experiments are shown, ***p < 0.001, **p < 0.01. Statistical analysis determined with two-tailed Student’s *t*-tests. **(G)** Quantification of the % percentage of anaphase cells having chromatin bridges. Means and SDs (error bars) from *n* = 3 biologically independent experiments are shown, ***p < 0.001, **p < 0.01. Statistical analysis determined with two-tailed Student’s *t*-tests. **(H)** Crystal violet staining of cell survival upon treatment with AF615 in RPE1 cells transfected with Geminin and CTRL siRNAs. **(I)** Survival curves of RPE1 cells treated with increased concentrations of compound AF615 (0, 11, 33, and 100 μΜ). *n* = 3 biologically independent experiments per compound concentration. Scale bars: 7 μm.

Several normal human cell lines are resistant to Geminin knockdown, in contrast to cancer cell lines in which extensive re-replication occurs (Zhu and DePamphilis, 2009; Huang et al., 2015). In accordance with previous reports, we were not able to identify any phosphorylation of H2AX or formation of 53BP1 foci in immortalized RPE1 cells transfected with Geminin siRNA (Supplementary Figures S6A–C). Since RPE1 seem to have a balanced ratio between CDT1 and Geminin proteins, we examined whether combined treatment of compound AF615 and Geminin siRNA may induce a phenotype similar to MCF7, U2OS and Saos-2 cells. To address this, RPE1 cells were cultured in the presence of increasing concentrations of compound AF615 and simultaneously transfected with Geminin siRNA. The activation of the DNA damage response was monitored through quantification of H2AX phosphorylation and 53BP1 foci formation using automated high-throughput microscopy (Figure 5A). Image analysis revealed that combined treatment of Geminin siRNA and compound AF615 (1.2 μΜ and higher concentrations) enhanced the phosphorylation of H2AX and the formation of 53BP1 foci compared to siCTRL and compound AF615 treated cells (Figures 5B,C). We next examined whether the elevated levels of DNA damage in cells with downregulation of Geminin and treatment with compound AF615 might lead to chromosomal instability and/or mitotic aberrations. The number of micronuclei and chromatin bridges in anaphase was quantified in RPE1 cells transfected with Geminin siRNA and treated with increasing concentrations of compound AF615. Cells cultured in the presence of AF615 (0.04–3.6 μΜ), which were also depleted for Geminin, accumulated a high number of micronuclei (Figures 5D,F) and chromatin bridges (Figures 5E,G). The combination of siGeminin and AF615 significantly reduced cell viability, preventing analysis of micronuclei formation and chromatin bridges at higher compound concentrations (11–33 μΜ). We then wished to investigate whether the elevated levels of DNA damage and the presence of chromosomal instability observed upon combination of Geminin siRNA-mediated knockdown and treatment with compound AF615, has an impact on cell survival. Crystal violet viability assays were performed in RPE1 cells depleted for Geminin and treated with increasing concentrations of chemical compound AF615. Reduced cell survival was observed in cells treated with 33 or 100 μΜ compound AF615 together with siGeminin, compared to control cells treated with siCTRL and compound AF615 (Figures 5H,I). Given that siGeminin does not eliminate completely the protein pools of Geminin in the cells, treatment with compound AF615 may inhibit the interaction of the reminiscent endogenous Geminin with CDT1, thereby inducing a phenotype similar to the MCF7, U2OS and Saos-2 cells, which harbor abnormal protein ratios of CDT1 and Geminin. In line with this, combined treatment of siGeminin and compound AF615 in MCF7 cells, which are sensitive to the compound, did not further enhance the phospohorylation of H2AX (Supplementary Figure S6D), suggesting that compound AF615 and siGeminin are epistatic.

## Discussion

Spatio-temporal regulation of DNA replication is tightly controlled through origin licensing. Aberrant expression of proteins participating in the licensing process promotes genomic instability, which in turn predisposes cells for malignant transformation (Petropoulos et al., 2019). Several studies provide evidence that cancer cells exhibit defective monitoring of licensing, and therefore proceed with DNA replication in cases of under- or over-licensing (Vaziri et al., 2003; Tatsumi et al., 2006; Ge et al., 2007; Ibarra et al., 2008; Matson et al., 2019) and are consequently more vulnerable to DNA damage caused by abnormal origin licensing (Melixetian et al., 2004; Ge et al., 2007; Ibarra et al., 2008; Nevis et al., 2009; Zhu and DePamphilis, 2009). Concomitantly, induction of DNA lesions is a common mechanism of action for many chemotherapeutic regimens (Cheung-Ong et al., 2013; Li et al., 2021). Therefore, deregulation of replication licensing constitutes an attractive target for the development of novel anticancer agents that will promote DNA damage and apoptosis in tumor cells. CDT1 and Geminin are central regulators of licensing, while their aberrant protein expression has been reported in various cancer-derived cell lines, as well as in different human tumor specimens (Karakaidos et al., 2004; Xouri et al., 2004; Bravou et al., 2005; Gonzalez et al., 2005; Tatsumi et al., 2006). Additionally, the differential response of CDT1 protein levels observed upon treatment with common genotoxic agents highlights its biological significance in the therapeutic approach to malignancies (Stathopoulou et al., 2012).

Here, we developed an *in-vitro* AlphaScreen™ high-throughput screening assay for the identification of small molecule inhibitors targeting the CDT1/Geminin protein complex. Protein interactions have been considered difficult to inhibit, mainly due to the large size of the contact surfaces involved and the lack of small molecule binding pockets. We introduced point mutations to CDT1 amino acid residues in order to generate a more easily disruptable target molecule. A diverse chemical library consisting of 23,360 compounds was screened and based on statistical criteria, AF615 was selected as the most specific and potent compound. Surface Plasmon Resonance analysis with the use of Geminin and CDT1 as individual proteins showed that AF615 compound inhibited binding of Geminin to CDT1, providing evidence that it can prevent the formation of the complex. Of note, further analysis using AlphaScreen™ technology revealed that the compound’s inhibitory effect occurs in a dose-dependent manner, thereby indicating its specificity. Other groups have also reported molecules such as coenzyme Q10 (Mizushina et al., 2008a), glycolipid SQDG (Mizushina et al., 2008b) and fatty acids (Mizushina et al., 2008c), which display inhibitory effects on CDT1/Geminin complex formation, by binding to CDT1. However, these studies do not provide evidence regarding cell specificity and biological mechanism of action, and the aforementioned molecules lack drug-like properties, something that restrains their development to potent pharmacological agents. Τhe compound AF615 described here represents the first drug-like small molecule inhibitor of CDT1/Geminin complex, that has been extensively characterized *in-vitro* and *ex-vivo* for its ability to regulate DNA replication.

For a small molecule inhibitor that targets an intracellular protein-protein interaction, being able to penetrate through the cell membrane is a prerequisite. This essential property is related to physicochemical parameters including molecular mass and total polar surface area (tPSA). It has been suggested that low molecular mass as well as tPSA value favors/facilitates penetration through the cell membrane (Ertl et al., 2000). In our case, compound AF615 had a molecular mass of 267.31 Da and a calculated tPSA of 114.47 Å^2^. The above parameters account for adequate membrane permeability. Indeed, by applying sensitized emission FRET in MCF7 cancer cells we could demonstrate the inhibitory effect of compound AF615 on Geminin-CDT1. Origin re-licensing accompanied by CDT1 overexpression or Geminin down-regulation, induces DNA re-replication in different cancer cell lines, promoting the activation of the DNA damage response, inhibition of DNA synthesis and cell cycle arrest in S or G2 phase (Klotz-Noack et al., 2012; Muñoz et al., 2017; Zhou et al., 2020). By employing high content imaging and automated image analysis, we identified activation of the DNA damage response in different cancer cell lines treated with compound AF615. Knock-down of CDT1 reduced the effect of compound AF615 in cancer cells, revealing that endogenous CDT1 is required for the mechanism of action of compound AF615. Moreover, cell cycle arrest and blockage of DNA synthesis in cells treated with AF615 was observed. This phenotype is consistent with the cellular effects evident upon origin re-licensing and re-replication in cancer cells, underlying that compound AF615 may have a similar effect by deregulating the complex CDT1/Geminin.

Deregulation of Geminin and CDT1 proteins results in different responses in normal versus cancer cells (Shreeram et al., 2002; Vaziri et al., 2003; Tatsumi et al., 2006; Zhu and DePamphilis, 2009). Chemical compound AF615 promoted an early S phase arrest in cancer cell lines (MCF7, U2OS, Saos-2), whereas it did not exhibit a profound effect on normal cell lines (MCF10A, RPE1). Moreover, cancer cells treated with compound AF615 showed reduced viability as opposed to normal cells. One plausible explanation for that observation would be that normal cells acquire redundant mechanisms (apart from Geminin-CDT1 protein interaction) for accurate CDT1 regulation to ensure normal replication licensing (Nishitani et al., 2006; Pozo and Cook, 2016; Zhou et al., 2020). A second explanation could be that cancer cells harbor abnormal protein ratios of CDT1 and Geminin, in which a partial inhibition of Geminin may be sufficient to induce activation of the DNA damage response and cell cycle arrest. Additionally, normal cells respond to licensing inhibitors by activating a licensing checkpoint and arresting temporarily in G1 phase (Matson et al., 2019; Mcintosh and Blow, 2012). On the contrary, the genetically unstable cancer cells might possibly have a defective licensing checkpoint (Shreeram et al., 2002; Nevis et al., 2009; Zimmerman et al., 2013; Gastl et al., 2020) while ectopic licensing of origins of replication outside G1 phase can cause genome re-replication (Klotz-Noack et al., 2012; Muñoz et al., 2017). A similar mechanism of action has been suggested for a family of arylquinolin-amines that was identified as inhibitors of replication licensing, by preventing the tight ORC-DNA interaction required for MCM2-7 loading onto chromatin (Gardner et al., 2017). Furthermore, neddylation inhibitors such as MLN4924 (pevonedistat) that block the activity of cullin-ring ubiquitin ligases (CRLs), lead to stabilization and accumulation of CDT1 (and CDC6), DNA re-replication and activation of the DNA damage response (Lin et al., 2010; Paiva et al., 2015; Lan et al., 2016; Walter et al., 2016; Vanderdys et al., 2018). Taken together, the above data suggest that normal cells retain intact convergent pathways to control replication licensing, whereas cancer cells lack these pathways and thus are more sensitive to agents targeting licensing components, as the CDT1/Geminin protein complex (Blow and Gillespie, 2008; Petropoulos et al., 2019).

Overall, this study provides a reliable and promising strategy for identification and characterization of small molecule chemical compounds that inhibit Geminin-CDT1 protein interaction. We strongly believe that, compound AF615 could contribute to investigating the functional significance of Geminin-CDT1 protein interaction in cellular processes such proliferation and differentiation. Moreover, we suggest that compound AF615 constitutes an ideal template for applying medicinal chemistry methods, in order to improve its pharmacological properties and become clinically useful as anticancer drug.

## Data Availability

The original contributions presented in the study are included in the article/Supplementary Material, further inquiries can be directed to the corresponding authors.
