# Supplementary figures and images for: Small Molecule Inhibitor Targeting CDT1/Geminin Protein Complex Promotes DNA Damage and Cell Death in Cancer Cells
Source: Front Pharmacol. 2022 Apr 25;13:860682. doi: 10.3389/fphar.2022.860682 (PMC9083542; doi:10.3389/fphar.2022.860682)

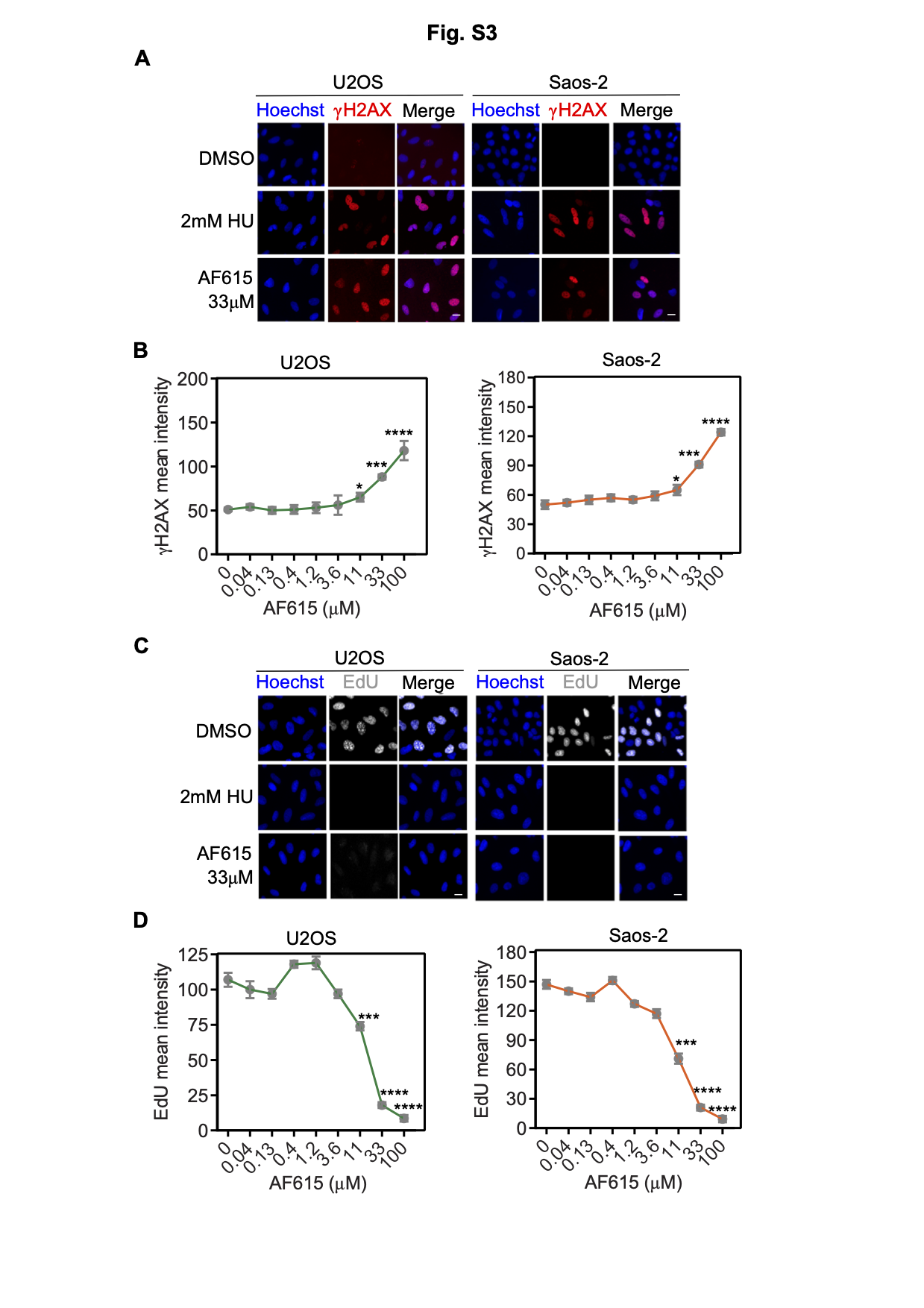

Supplement: Supplementary file 1 [file Image3.tiff]

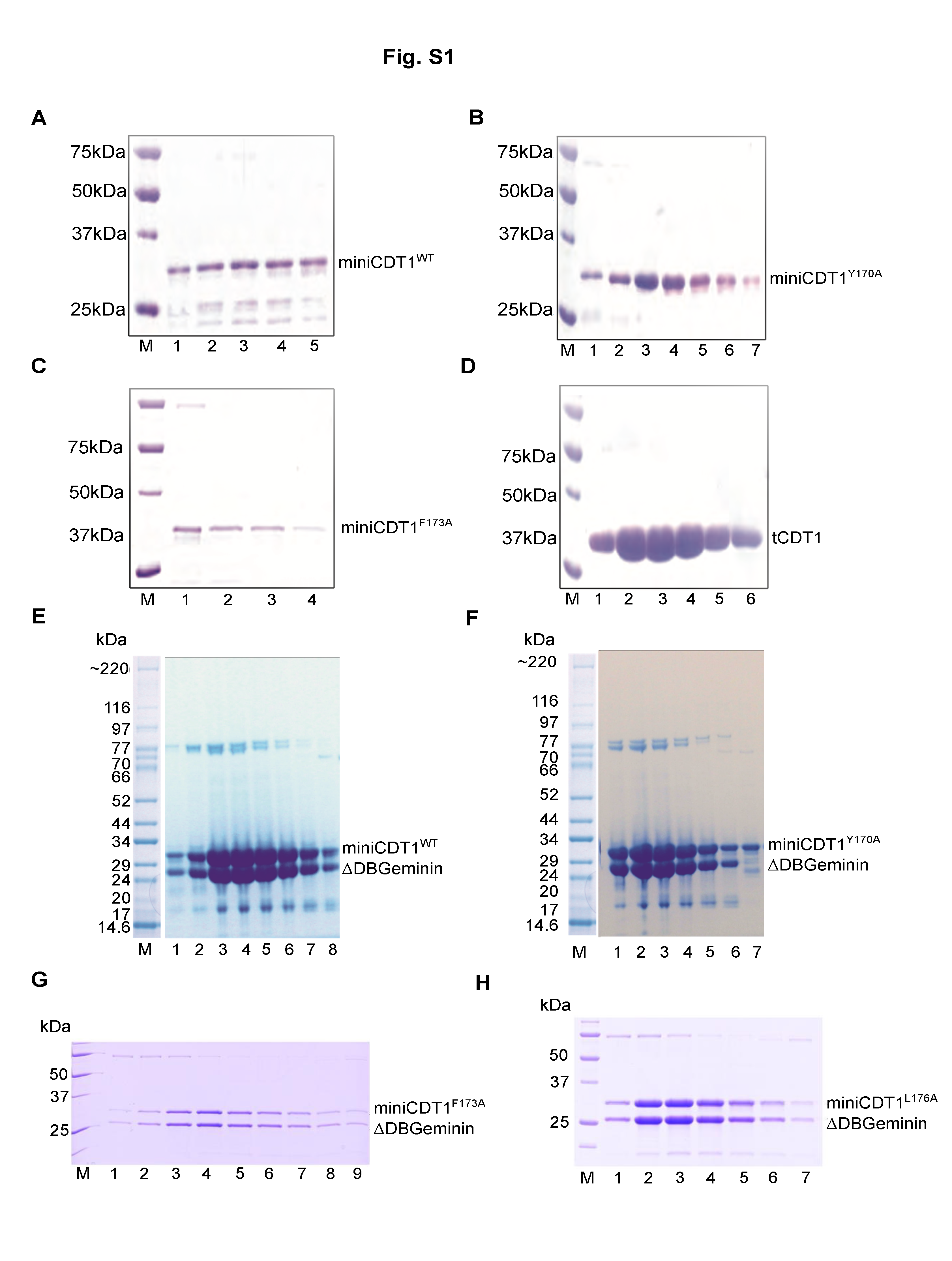

Supplement: Supplementary file 2 [file Image1.tiff]

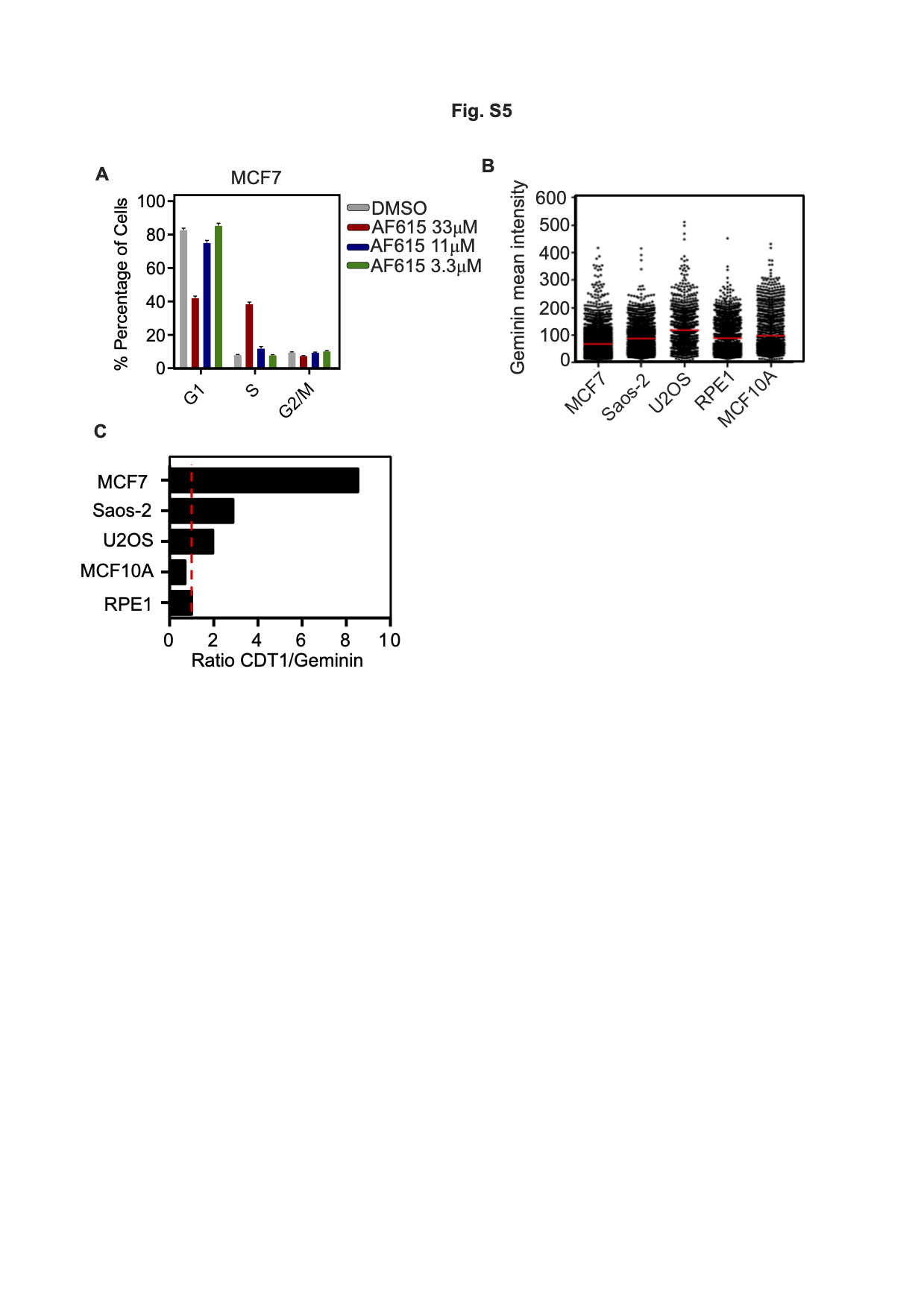

Supplement: Supplementary file 4 [file Image5.tiff]

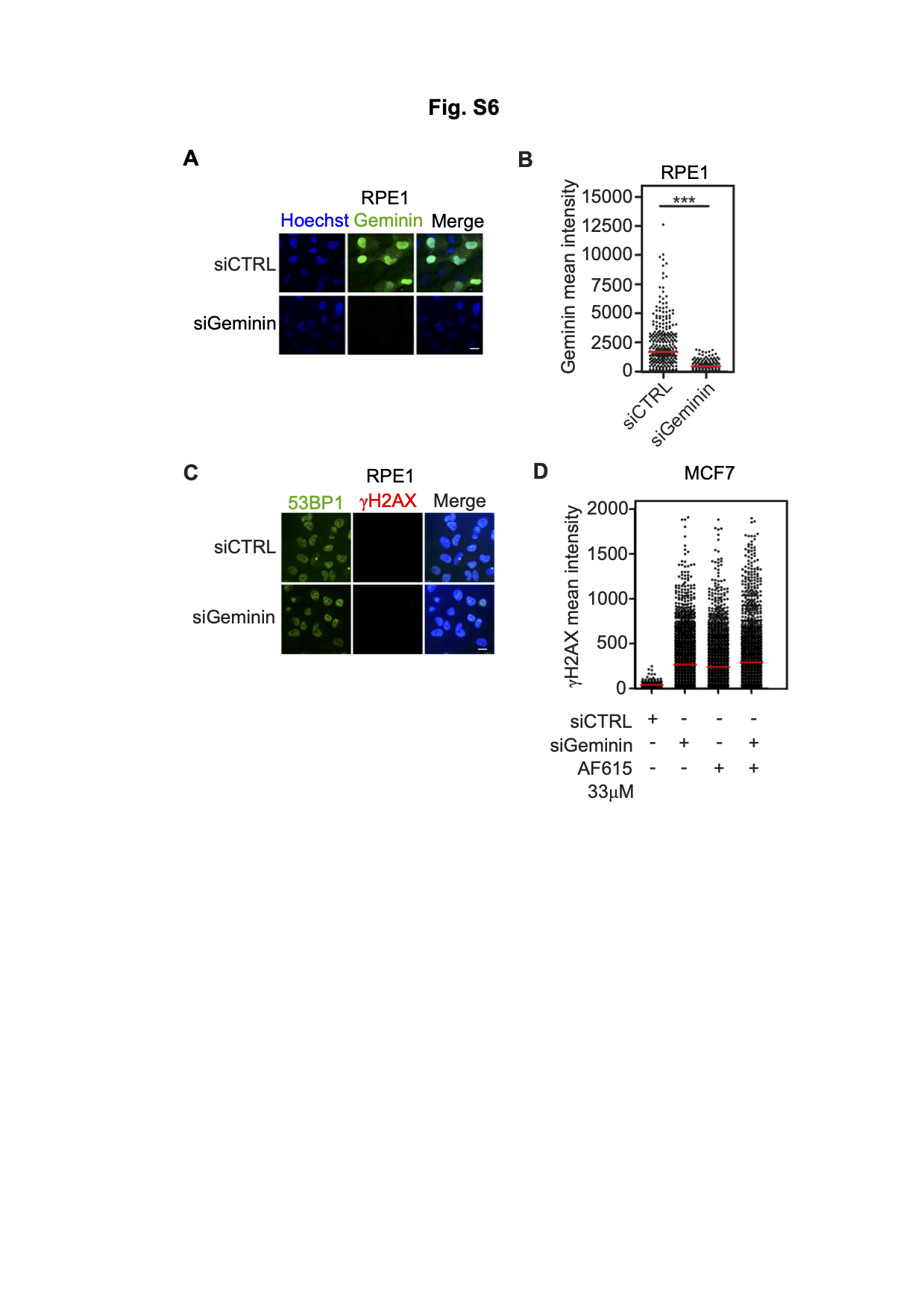

Supplement: Supplementary file 5 [file Image6.tiff]

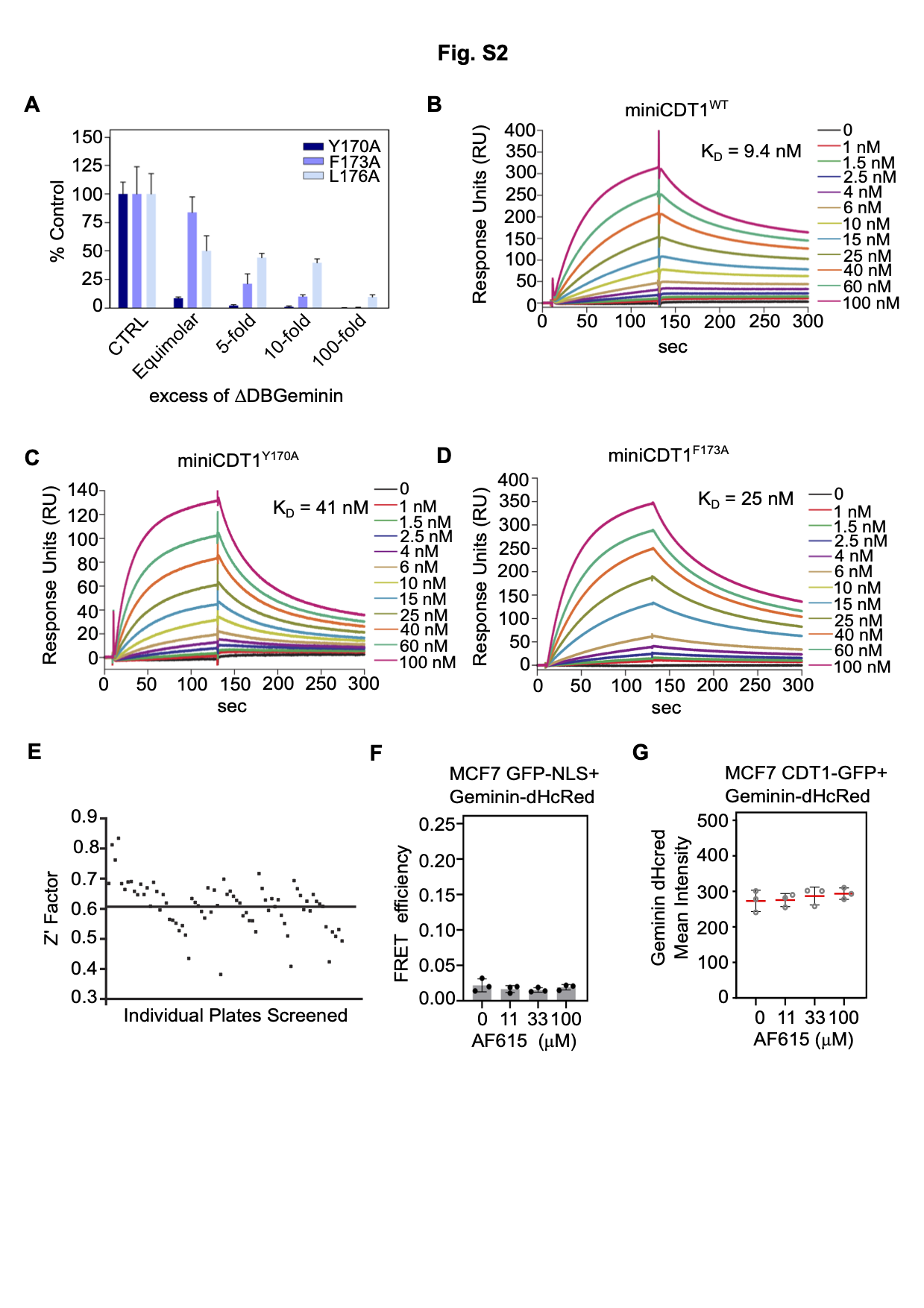

Supplement: Supplementary file 6 [file Image2.tiff]

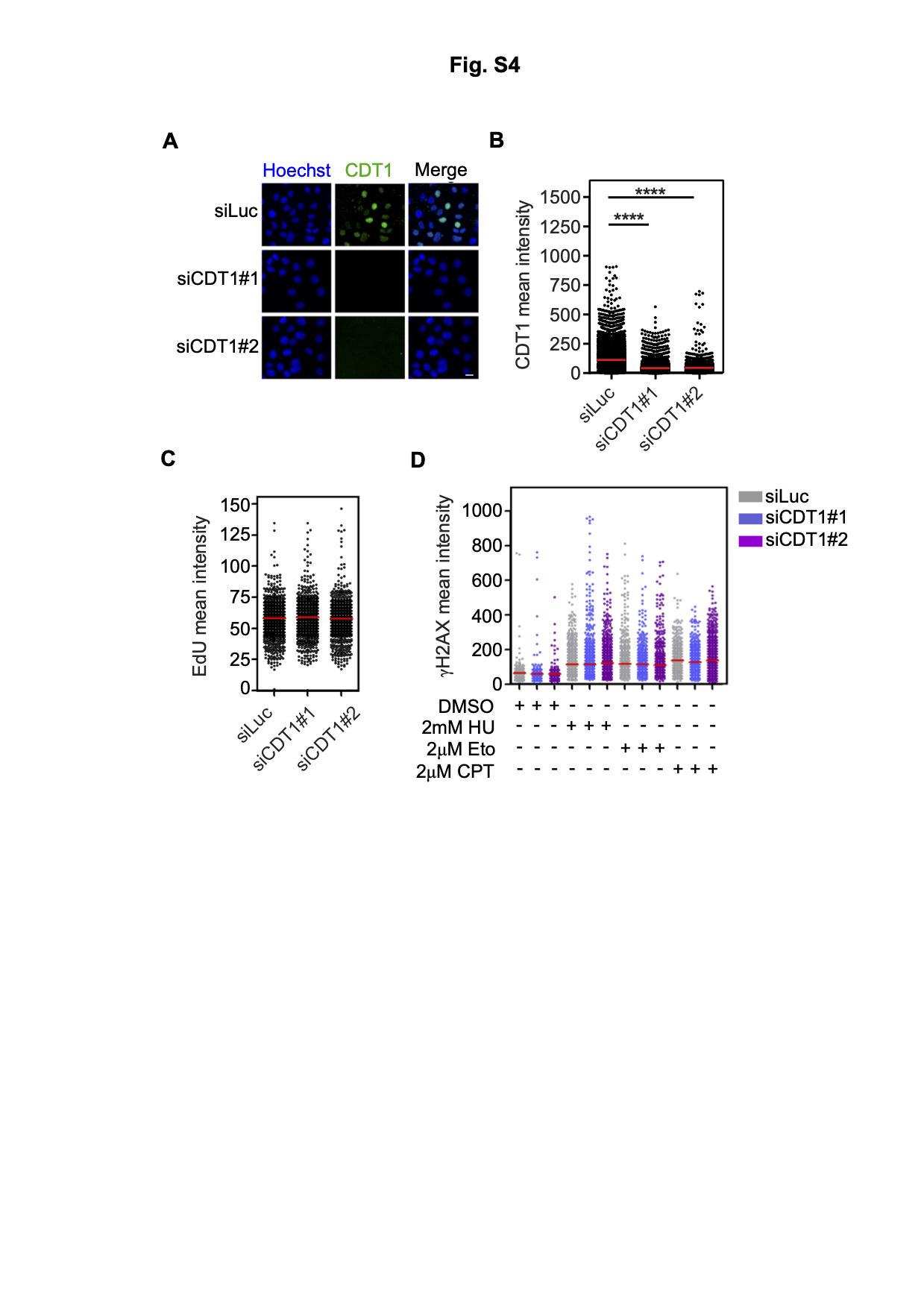

Supplement: Supplementary file 7 [file Image4.tiff]
