# Supplementary material for: Small Molecule Inhibitor Targeting CDT1/Geminin Protein Complex Promotes DNA Damage and Cell Death in Cancer Cells
Source: Front Pharmacol. 2022 Apr 25;13:860682. doi: 10.3389/fphar.2022.860682 (PMC9083542; doi:10.3389/fphar.2022.860682)
Supplement: Supplementary file 3 [file Table1.docx]

**Supplemental Information**

**Title: Small molecule inhibitor targeting CDT1/Geminin protein complex promotes DNA damage and cell death in cancer cells.**

Nikolaos Karantzelis^#^, Michalis Petropoulos^#^, Valeria De Marco, David A. Egan, Alexander Fish, Evangelos Christodoulou, David W. Will, Joe D. Lewis, Anastassis Perrakis, Zoi Lygerou and Stavros Taraviras

# These authors contributed equally to this work.

**PDF File contains:**

**Supplementary Figures 1-6**

**Supplementary Figure Legends 1-6**

**Supplementary figure 1:** Integrity and purity of the CDT1 protein constructs and Geminin-CDT1 complexes used for surface plasmon resonance analysis and AlphaScreen^TM^ assay respectively, assessed by SDS-PAGE. **a,** miniCDT1 Wild Type (WT), **b**, miniCDT1^Y170A^, **c,** miniCDT1^F173A^ , **d,** tCDT1, **e**, Flag-ΔDBGeminin/His-miniCDT1^WT^ complex, **f**, Flag-ΔDBGeminin/His-miniCDT1^Y170A^ complex, **g**, Flag-ΔDBGeminin/His-miniCDT1^F173A^ complex and **h**, Flag-ΔDBGeminin/His-miniCDT1^L176A^ complex were all bacterially expressed and purified by Immobilized Metal Affinity Chromatography (IMAC) followed by size-exclusion chromatography. Fractions of the last purification step, after gel filtration, as indicated by the numbers depicted. M: Molecular weight protein marker.

**Supplementary figure 2: a,** Excess of non-tagged free ΔDBGeminin was used to disrupt each of the three Flag-ΔDBGeminin/His-miniCDT1 mutant complexes (CDT1 residues Y170A, F173 and L176 were mutated to alanine). Control levels (%) of maximal AlphaScreen^TM^ signal (100%) were determined in the absence of non-tagged free ΔDBGeminin. Values shown are normalized to the control and y-axis represents the percentage reduction of the maximal AlphaScreen^TM^ signal. Data are expressed as means and error bars indicate standard deviations for triplicate measurements. **b,** Surface plasmon resonance binding curves of Geminin His-miniCDT1 WT, **c,** His-miniCDT1^Y170A^ mutant and **d,** His-miniCDT1^F173A^ mutant. MiniCDT1 protein constructs were immobilized to a Ni NTA Biacore chip. The binding of CDT1 to Geminin was detected by surface plasmon resonance signal and is indicated in response units. Twelve different concentrations of Geminin (curve 1, 0 nM; curve 2, 1 nM; curve 3, 1.5 nM; curve 4, 2.5 nM; curve 5, 4 nM; curve 6, 6 nM; curve 7, 10 nM; curve 8, 15 nM; curve 9, 25 nM; curve 10, 40 nM; curve 11, 60 nM; curve 12, 100 nM) were injected over the chip. The dissociation constant (K_D_) for the Geminin-miniCDT1 WT interaction was about 9 nM, while binding to miniCDT1^Y170A^ mutant exhibited an affinity reduced by about a factor of four (K_D_=41 nM). MiniCDT1^F173A^ mutant showed an intermediate affinity (K_D_=25 nM). Binding curves were recorded, using the empty flow cell as reference. **e,** Evaluation of Z’ factor. Dots represent Z’ values for each screening plate, while solid line represents average Z’ average value for the entire HTS. **f,** Graphical representation of FRET efficiency in MCF7 GFP-NLS cells transfected with GeminindHcRed in the presence or absence of increasing concentrations (0, 11, 33, 100 μΜ) of compound AF615 for 24 hours. Dots represent each mean of three independent biological replicates and the error bars represent standard deviation. **g,** Graphical representation of Geminin-dHcred mean intensity in MCF7-CDT1 GFP stable cells treated with increasing concentrations (0, 11, 33, 100 μΜ) of compound AF615 for 24 hours.

**Supplementary figure 3:** Compound AF615 induces DNA damage and inhibits DNA synthesis in U2OS and Saos-2 cell lines. **a,** Representative images of U2OS and Saos2 cells treated with 33 μΜ compound AF615 for 24 hours and immunostained for γH2AX. Cell nuclei were counterstained with Hoechst. **b,** Quantification of γΗ2ΑΧ mean intensity in U2OS and Saos-2 cells treated with increasing concentrations of AF615 for 24 hours. Graphs represent the mean γΗ2ΑΧ intensity of *n*=3 biological independent experiments per compound AF615 concentration**. c,** Representative images of U2OS and Saos2 cells pulsed labeled with EdU and treated with 33 μΜ compound AF615 for 24 hours. Cell nuclei were counterstained with Hoechst. **d,** Quantification of mean EdU intensity in U2OS and Saos-2 cells treated with increasing concentrations of AF615 for 24 hours. Graphs depict the mean EdU intensity of *n*=3 biological independent experiments per compound AF615 concentration**. ******p<*0.0001, ****p* <0.001, ***p* < 0.01. Statistical analysis determined with two-tailed Student’s t-tests. Scale bars: 7 μm.

**Supplementary figure 4: a,** Representative images of MCF7 transfected with siLuciferase, siCDT1#1 and siCDT1#2 and immunostained for CDT1. Nuclei were counterstained with Hoechst. **b,** Scatter plot depicts per cell CDT1 mean intensity. Data are representative of *n*=3 independent experiments. **c,** Graphical representation of EdU mean intensity of MCF7 cells transfected with siLuciferase, siCDT1#1, siCDT1#2. **d,** Quantification of γΗ2ΑΧ mean intensity of MCF7 cells transfected with siLuciferase, siCDT1#1, siCDT1#2 and treated with 2 mM HU, or 2 μΜ Εtoposide or 2 μΜ Camptothecin for 24 hours. Statistical analysis was performed using Mann-Whitney Student’s t-tests. *****p* < 0.0001. Scale bar: 7 μm.

**Supplementary figure 5: a,** Quantification of the cell cycle distribution of MCF7 cells treated with 3.3, 11, 33 μΜ compound AF615. **b,** Graphical representation of Geminin mean intensity in MCF7, Saos-2, U2OS, RPE1 and MCF10A cell lines. **c,** Quantification of the ratio of CDT1/Geminin protein levels in MCF7, U2OS, Saos-2, RPE1 and MCF10A cells.

**Supplementary figure 6: a,** Representative images of RPE1 cells transfected with siCTRL or siGeminin and immunostained for Geminin. Nuclei counterstained with Hoechst. **b,** Quantification of Geminin mean intensity of RPE1 cells transfected with siCTRL and siGeminin. **c,** Representative images of RPE1 cells transfected with siCTRL or siGeminin and immunostained for γΗ2ΑΧ and 53BP1. Nuclei counterstained with Hoechst. **d,** Quantification of γΗ2ΑΧ mean intensity of MCF7 cells transfected with siCTRL or siGeminin and treated with DMSO or 33 μΜ compound AF615 for 24 hours. Statistical analysis was performed using Mann-Whitney Student’s t-tests. ****p* < 0.001. Scale bars: 7 μm.
